# ﻿Unexpected richness and distinct patterns of *Morchella* (*Ascomycota*) species diversity in Chongqing, a notable “Furnace City”: unveiling rich diversity in hot regions

**DOI:** 10.3897/imafungus.16.152685

**Published:** 2025-08-12

**Authors:** Qin Qin, Yan-Fei Teng, Wen Shu Hu, Jing-Yi Wei, Zhong-Dong Yu, Ping Du, Xiao-Yan Zhang, Xia Guo, Meng-Qian Chen, Wei Wei, Xi-Hui Du

**Affiliations:** 1 College of Life Sciences, Chongqing Normal University, Chongqing 401331, China Chongqing Normal University Chongqing China; 2 Chongqing Academy of Agricultural Sciences, Chongqing 401329, China Chongqing Academy of Agricultural Sciences Chongqing China; 3 College of Forestry, Northwest A&F University, Yangling 712100, Shaanxi, China Northwest A&F University Yangling China; 4 School of Advanced Agriculture and Bioengineering, Yangtze Normal University, Chongqing 408100, China Yangtze Normal University Chongqing China

**Keywords:** *Morchellachinensis* new taxon, *Morchelladiversa* new taxon, *Morchellaeoa* new taxon, *Morchellahuoguo* new taxon, *Morchellamontana* new taxon, *Morchellanipponensis* new record, *Morchellauniversitatis* new taxon, vegetation impact

## Abstract

*Morchella* species, commonly known as true morels and being cold-preferring fungi, are esteemed for their distinctive flavor as well as significant economic and prominent research value. Chongqing, located in southwestern China and renowned as the “Furnace City” due to its distinctive climate and extremely high summer temperatures, spans an area of 82,400 square kilometers with complex geographical topography, yet has lacked a comprehensive survey of true morels (*Morchella*) so far. From 2017 to 2024, we conducted extensive field surveys across 13 districts and counties within Chongqing, resulting in the collection of over 1,000 wild morel samples. Through a combination of multi-gene phylogenetic analysis and microscopic morphological observations, we uncovered a surprisingly high level of species richness of *Morchella* in Chongqing, identifying 13 species in the Esculenta clade (yellow morels) and three species in the Elata clade (black morels), including six newly described species: *Morchellachinensis***sp. nov.**, *M.diversa***sp. nov.**, *M.eoa***sp. nov.**, *M.huoguo***sp. nov.**, *M.montana***sp. nov.**, and *M.universitatis***sp. nov.** Notably, *M.nipponensis*, previously documented only in Japan, is reported for the first time in China. Furthermore, significant divergence in species diversity between the Esculenta and Elata clades of *Morchella* has been observed in Chongqing, which is strongly influenced and shaped by the prevailing vegetation. The distribution pattern of *Morchella* species suggests that the impact of high summer temperatures on species diversity in Chongqing is partially mitigated by locally diverse mountainous habitats. In addition, the habitats of *M.diversa* and *M.universitatis*, the two most widely distributed species in Chongqing, exhibit higher vegetation diversity, suggesting that *Morchella* species with greater habitat adaptability tend to have broader geographic ranges. This study provides valuable insights into the species diversity and distribution pattern of *Morchella*, particularly in a region with unique climatic and ecological conditions, and highlights the need for further study into the correlation between vegetation and *Morchella* species.

## ﻿Introduction

True morels (*Ascomycota*, *Morchella*) are renowned as rare edible fungi widely distributed across the Northern Hemisphere (Dahlstrom et al. 2000; O’Donnell et al. 2011; Du et al. 2012a, 2015; Loizides et al. 2021), rich in protein, vitamins, minerals, and other nutrients that offer numerous health benefits (Li et al. 2023; Tian et al. 2023). These fungi are highly valued for their exceptional flavor, unique texture, and significant economic importance in global markets (Tietel and Masaphy 2018). As a result, *Morchella* has been the focus of extensive research, particularly in areas such as species diversity, biogeography, reproduction modes, fungal disease, artificial cultivation, and more (Hervey et al. 1978; Dahlstrom et al. 2000; Du et al. 2012a, 2016, 2017, 2023; Hao et al. 2019; Liu et al. 2019; Ali et al. 2021; Du and Yang 2021; Cao et al. 2022; Deng et al. 2022; Meng et al. 2022, 2024; Hu et al. 2024; Mifsud et al. 2024; Tao et al. 2025). Molecular studies have established *Morchella* as a species-rich genus, encompassing approximately 80 phylospecies globally and divided into three well-defined clades: the Rufobrunnea clade (blushing morels, sect. Rufobrunnea), the Esculenta clade (yellow morels, sect. Morchella), and the Elata clade (black morels, sect. Distantes) (Guzmán and Tapia 1998; Pilz et al. 2007; Clowez 2010; Taşkın et al. 2010, 2012, 2015; O’Donnell et al. 2011; Du et al. 2012a, 2012b, 2019; Richard et al. 2015; Loizides et al. 2016, 2021). Many species in *Morchella* exhibit strong patterns of continental endemism and provincialism (O’Donnell et al. 2011; Du et al. 2012a; Loizides et al. 2016, 2021) and demonstrate obvious phylogenetic niche conservation, typically associated with either temperate deciduous forests or coniferous forests (Kuo et al. 2012; Du et al. 2012a, 2015; Baran and Boroń 2017). East Asia, particularly China, has been recognized as the center of modern species diversity and distribution of *Morchella* (Du et al. 2012a, 2014, 2015), a view further supported recently by the identification of six new species and the documentation of two new records identified from China (Du et al. 2019). So far, China has recorded a total of 16 phylospecies within the Elata clade and 21 phylospecies within the Esculenta clade.

Chongqing Municipality, located in southwestern China and covering an area of 82,400 square kilometers (Zhang et al. 2024), is well known as a “Furnace City” due to its extremely hot summers (Zhang et al. 2008; He et al. 2013; Huang et al. 2022) and is characterized by a subtropical monsoon climate with diverse ecological environments (Dai 2000; Liu 2000; Liu et al. 2010; Ding et al. 2013; Zhang et al. 2024). Moreover, Chongqing, also known as the “Mountain City,” lies to the east of the Sichuan Basin, adjacent to the Qinghai-Tibet Plateau in the west and the Yunnan-Guizhou Plateau in the south, and is predominantly encircled by mountains, with limited expanses of plains and hills in the western and southwestern areas. The Daba Mountain range extends across northern areas of Chongqing, such as Chengkou and Wuxi, whereas the Wuling Mountain dominates the southeast, spanning Qianjiang, Nanchuan, Wulong, Shizhu, Youyang, Fengdu, and Pengshui (Dai 2000; Tang et al. 2022; Liu et al. 2024; Yang et al. 2024). The unique geography exposes Chongqing to the East Asian and Indian monsoons and Qinghai-Tibet Plateau circulation, which restrict air movement and trap heat, earning it the title of one of China’s “Four Furnace Cities” (Zhang et al. 2008; He et al. 2013; Huang et al. 2022). In addition, Chongqing is located at the intersection of the Sino-Japanese Forest subkingdom and the Sino-Himalayan Forest subkingdom, placing it within the subtropical evergreen broadleaf and coniferous forest region (Liu 2000; Ding et al. 2013).

Despite possessing a unique climate and complex geography, the species diversity of *Morchella* in Chongqing has yet to be sufficiently explored and has long been overlooked due to the local extremely high temperatures, with only three species (*Mes*-19, *Mes*-23, and *Mel*-21) previously reported (Du et al. 2012a; Cai et al. 2020). Given Chongqing’s ecological complexity and floristic diversity, we hypothesize that comprehensive sampling and in-depth investigation of *Morchella* from this region will yield valuable insights into the diversity and distribution of the genus. Towards this end, we conducted an extensive eight-year sampling survey across 13 districts and counties in Chongqing, resulting in the collection of over 1,000 samples. Using the genealogical concordance phylogenetic species recognition criterion (GCPSR; Dettman et al. 2003) and morphological observations, we seek to address the following key questions: (1) What is the species diversity of *Morchella* in Chongqing, and how does it compare to that in other regions in China? (2) What are the distribution patterns of *Morchella* species in Chongqing? (3) Do climatic and ecological factors, such as high temperature and vegetation type, influence species diversity and distribution patterns of *Morchella* species in this region?

## ﻿Materials and methods

### ﻿Sampling

**Figure 1.**
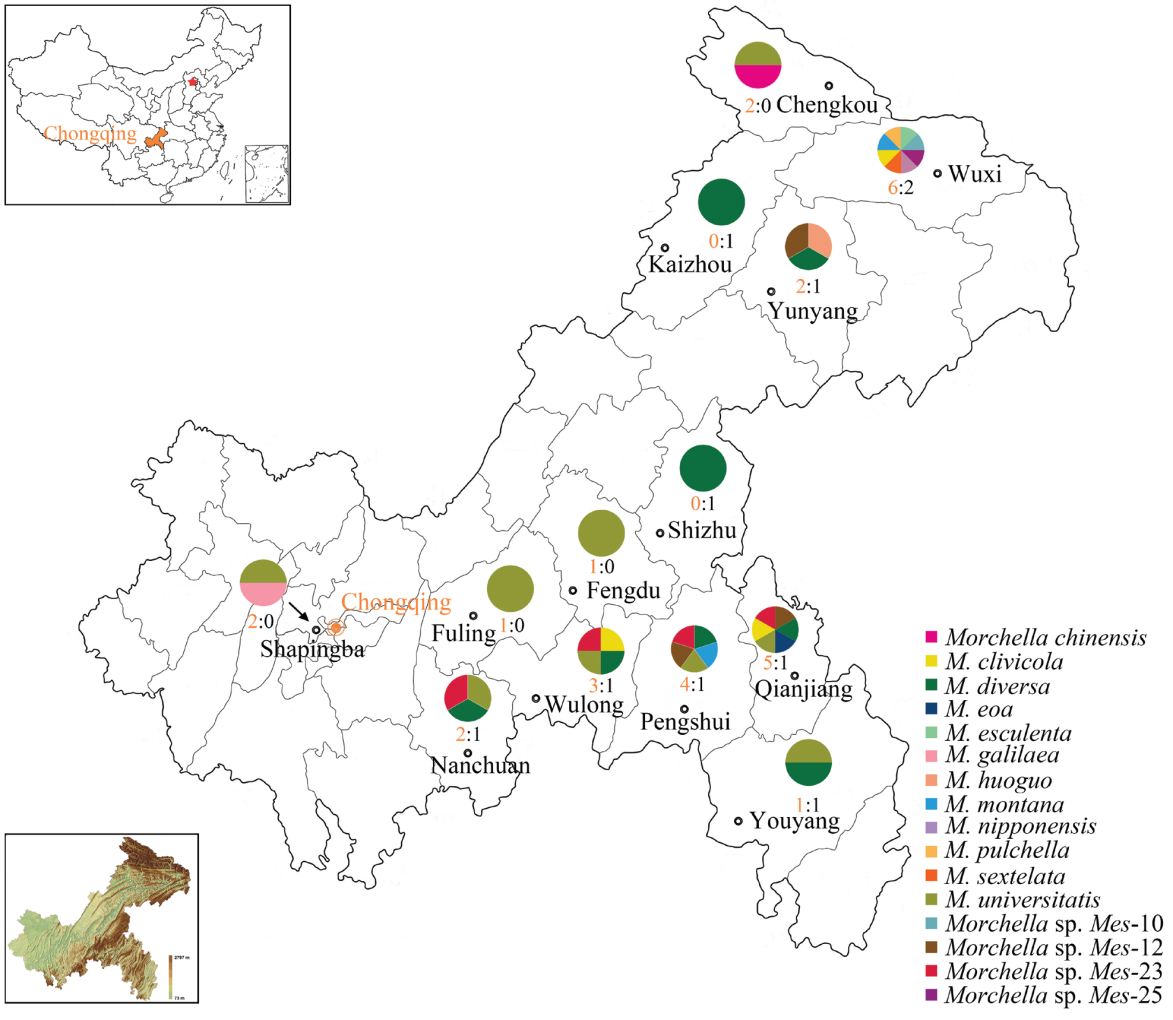
Map of Chongqing, China, identifying collection sites and species distribution of *Morchella*. From the 13 districts and counties in Chongqing, 13 species from the Esculenta clade (yellow morels) and 3 species from the Elata clade (black morels) were collected between 2017 and 2024. The differently colored sections in the pie charts represent the different species identified in each corresponding district or county, while the numbers below the pies indicate the ratio of species from the Esculenta clade to those from the Elata clade in that area. The top left picture is the map of China showing the location of Chongqing, while the bottom left picture depicts the complex geographical topology of Chongqing.

**Figure 2.**
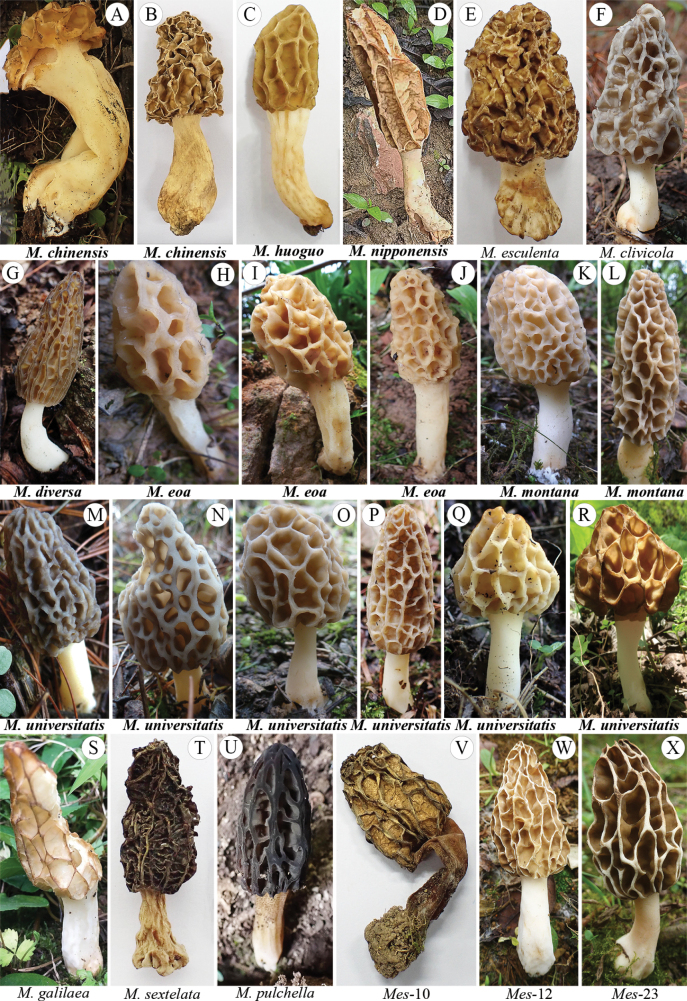
Morphological diversity of representative ascocarps of *Morchella* species collected in Chongqing, China. These ascocarps belong to 12 species from the Esculenta clade (A-F, H-S, V-X, yellow morels) and three from the Elata clade (G, T-U, black morels). A, B. *M.chinensis*. C. *M.huoguo*. D. *M.nipponensis*. E. *M.esculenta*. F. *M.clivicola*. G. *M.diversa*. H–J. *M.eoa*. K–L. *M.montana*. M–R. Juvenile and mature ascocarps of *M.universitatis*. S. *M.galilaea*. T. *M.sextelata*. U. *M.pulchella*. V. *Mes*-10. W. *Mes*-12. X. *Mes*-23. Species names of the six newly described and one newly recorded species are highlighted in bold in the figure.

**Figure 3.**
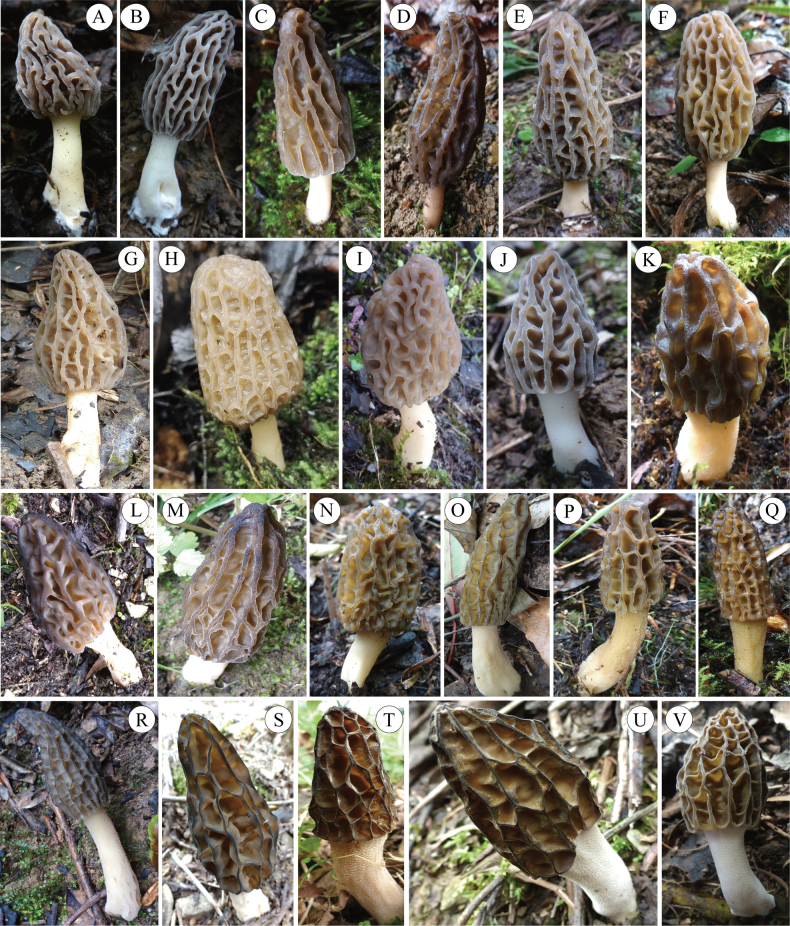
Morphological variations of *M.diversa* ascocarps at different developmental stages and in various environments. All specimens were collected in Chongqing, China.

Between 2017 and 2024, over 1,000 morel samples were collected from 13 districts and counties in Chongqing during the fruiting season each year (Figs 1–3). The specimens were photographed in the field, and information about their dominant vegetation and GPS coordinates—including altitude, latitude, and longitude—was recorded (Table 1). The specimens were then dried with silica gel or in a portable dryer, transported to the laboratory at the end of each field trip, and finally deposited in the Fungal Herbarium of Chongqing Normal University (FCNU), Chongqing.

**Table 1.** Details of the 84 *Morchella* specimens newly collected for multi-gene phylogenetic analysis in this study. Species names of the newly described and newly recorded taxa are indicated in bold.

Species	Specimen voucher	Location	Collection date	Dominant vegetation	GPS coordinates	GenBank accession number			
						ITS	EF1-a	RPB1	RPB2
***Morchellachinensis***s	FCNU1326	Chengkou	2024.4.26	Weeds	108.9779°E-31.8925°N (DD)-ca 1560 m	PV156392	PV227241	PV227235	PV204696
***M.chinensis***	FCNU1445	Ankang^*^	2017.5.15	*Quercus* sp.	109.2667°E-34.2500°N (DD)-ca 1500 m	PV156431	PV227238	PV227232	PV204693
***M.chinensis***	FCNU1446	Ankang^*^	2017.5.15	*Juglans* sp.	109.2667°E-34.2500°N (DD)-ca 1500 m	PV156432	PV227239	PV227233	PV204694
***M.chinensis***	FCNU1447	Xi’an^*^	2017.5	*Castanea* sp.	110.3167°E-35.1167°N (DD)-Unknown	PV156433	PV227240	PV227234	PV204695
***M.huoguo***	FCNU1335	Yunyang	2024.4.14	*Pinus* sp.	108.9067°E-30.4672°N (DD)-ca 1320 m	PV156397	PV227236	PV227230	PV204691
***M.huoguo***	FCNU1337	Yunyang	2024.4.14	*Pinus* sp.	108.9067°E-30.4672°N (DD)-ca 1320 m	PV156399	PV227237	PV227231	PV204692
***M.eoa* (*Morchella* sp. Mes-15)**	FCNU1364	Qianjiang	2024.4.7	*Pyrus* sp.	108.8019°E-29.4601°N (DD)-ca 490 m	PV156412	PV227270	PV227209	PV175872
***M.eoa* (*Morchella* sp. Mes-15)**	FCNU1365	Qianjiang	2024.4.7	*Pyrus* sp.	108.8019°E-29.4601°N (DD)-ca 490 m	PV156413	PV227271	PV227217	PV175875
***M.eoa* (*Morchella* sp. Mes-15)**	FCNU1366	Qianjiang	2024.4.7	*Pyrus* sp.	108.8019°E-29.4601°N (DD)-ca 490 m	PV156414	PV227273	PV227193	PV175873
***M.universitatis* (*Morchella* sp. Mes-19**)	FCNU1121	Shapingba	2017.4.10	*Buxus* sp.	106.3015°E-29.6155°N (DD)-ca 280 m	PV156439	PV227265	PV227203	PV175879
***M.universitatis* (*Morchella* sp. Mes-19)**	FCNU1122	Youyang	2017.4.3	Unknown	108.4547°E-28.4129°N (DD)-ca 930 m	PV156440	PV227255	PV227204	PV175864
***M.universitatis* (*Morchella* sp. Mes-19)**	FCNU1201	Pengshui	2023.4.9	Weeds	108.0763°E-29.2412°N (DD)-ca 930 m	PV156367	PV227252	PV227198	PV175857
***M.universitatis* (*Morchella* sp. Mes-19)**	FCNU1202	Pengshui	2023.4.9	Weeds	108.0763°E-29.2412°N (DD)-ca 930 m	PV156368	PV227257	PV227205	PV175861
***M.universitatis* (*Morchella* sp. Mes-19)**	FCNU1215	Wulong	2023.4.10	*Kalopanax* sp.	108.0037°E-29.2402°N (DD)-ca 1160 m	PV156375	PV227253	PV227199	PV175876
***M.universitatis* (*Morchella* sp. Mes-19)**	FCNU1318	Chengkou	2024.4.26	*Rheum* sp.	108.9779°E-31.8925°N (DD)-ca 1560 m	PV156389	PV227258	PV227214	PV175858
***M.universitatis* (*Morchella* sp. Mes-19)**	FCNU1319	Chengkou	2024.4.26	*Rheum* sp.	108.9779°E-31.8925°N (DD)-ca 1560 m	PV156390	PV227254	PV227215	PV175859
***M.universitatis* (*Morchella* sp. Mes-19)**	FCNU1325	Chengkou	2024.4.26	*Rheum* sp.	108.9779°E-31.8925°N (DD)-ca 1560 m	PV156391	PV227260	PV227216	PV175860
***M.universitatis* (*Morchella* sp. Mes-19)**	FCNU1333	Fengdu	2024.4.4	*Cupressus* sp.	107.6703°E-30.2064°N (DD)-ca 500 m	PV156395	PV227261	PV227200	PV175862
***M.universitatis* (*Morchella* sp. Mes-19)**	FCNU1334	Fengdu	2024.4.4	*Cupressus* sp.	107.6703°E-30.2064°N (DD)-ca 500 m	PV156396	PV227262	PV227201	PV175863
***M.universitatis* (*Morchella* sp. Mes-19)**	FCNU1367	Qianjiang	2024.4.8	*Pinus* sp.	108.6217°E-29.1378°N (DD)-ca 1080 m	PV156415	PV227263	PV227206	PV175877
***M.universitatis* (*Morchella* sp. Mes-19)**	FCNU1368	Qianjiang	2024.4.8	*Pinus* sp.	108.6217°E-29.1378°N (DD)-ca 1080 m	PV156416	PV227259	/	PV175878
***M.universitatis* (*Morchella* sp. Mes-19)**	FCNU1381	Qianjiang	2024.4.8	*Pinus* sp.	108.6204°E-29.1353°N (DD)-ca 1190 m	PV156420	PV227256	PV227202	PV175880
***M.universitatis* (*Morchella* sp. Mes-19)**	FCNU1444	Fuling	2023.3.31	Weeds	107.1536°E-29.4476°N (DD)-ca 290 m	PV156430	PV227264	/	/
***M.montana* (*Morchella* sp. Mes-20)**	FCNU1206	Pengshui	2023.4.9	*Castanopsis* sp.	108.0795°E-29.2365°N (DD)-ca 1010 m	PV156371	PV227277	PV227221	PV175868
***M.montana* (*Morchella* sp. Mes-20)**	FCNU1207	Pengshui	2023.4.9	*Castanopsis* sp.	108.0795°E-29.2365°N (DD)-ca 1010 m	PV156372	PV227266	PV227222	/
***M.montana* (*Morchella* sp. Mes-20)**	FCNU1328	Wuxi	2024.4.27	*Raphanus* sp.	109.0239°E-31.6597°N (DD)-ca 1880 m	PV156394	PV227276	PV227223	PV175852
***M.montana* (*Morchella* sp. Mes-20)**	FCNU1350	Wuxi	2024.4.14	Weeds	109.8023°E-31.4714°N (DD)-ca 1670 m	PV156408	PV227274	PV227224	PV175869
***M.montana* (*Morchella* sp. Mes-20)**	FCNU1357	Wuxi	2024.4.25	Weeds	109.8220°E-31.5080°N (DD)-Unknown	PV156410	PV227278	PV227225	PV175870
***M.diversa* (*Morchella* sp. *Mel*-21**)	FCNU1143	Youyang	2018.3.28	Bamboo	108.4828°E-28.3975°N (DD)-ca 960 m	PV156434	PV227305	PV211892	PV175827
***M.diversa* (*Morchella* sp. *Mel*-21)**	FCNU1144	Youyang	2018.3.28	Bamboo	108.4828°E-28.3975°N (DD)-ca 960 m	PV156435	PV227287	PV211893	PV175828
***M.diversa* (*Morchella* sp. *Mel*-21)**	FCNU1145	Youyang	2018.3.28	Bamboo	108.4828°E-28.3975°N (DD)-ca 960 m	PV156436	PV227288	PV211905	PV175829
***M.diversa* (*Morchella* sp. *Mel*-21)**	FCNU1147	Youyang	2018.3.28	Bamboo	108.4828°E-28.3975°N (DD)-ca 960 m	PV156437	PV227304	PV211906	PV175830
***M.diversa* (*Morchella* sp. *Mel*-21)**	FCNU1148	Youyang	2018.3.28	Bamboo	108.4828°E-28.3975°N (DD)-ca 960 m	PV156438	PV227289	PV211907	PV175831
***M.diversa* (*Morchella* sp. *Mel*-21)**	FCNU1149	Pengshui	2023.3.22	Bamboo	107.9965°E-29.1861°N (DD)-ca 1530 m	PV156441	PV227284	PV211908	PV175832
***M.diversa* (*Morchella* sp. *Mel*-21)**	FCNU1155	Pengshui	2023.3.22	Bamboo	108.0216°E-29.1976°N (DD)-ca 1450 m	PV156442	PV227290	PV211894	PV175833
***M.diversa* (*Morchella* sp. *Mel*-21)**	FCNU1157	Pengshui	2023.3.22	Bamboo	108.0216°E-29.1976°N (DD)-ca 1450 m	PV156443	PV227291	PV211895	PV175834
***M.diversa* (*Morchella* sp. *Mel*-21)**	FCNU1165	Pengshui	2023.3.23	*Quercus* sp.	108.0038°E-29.2147°N (DD)-ca 1290 m	PV156444	PV227292	PV211910	PV175835
***M.diversa* (*Morchella* sp. *Mel*-21)**	FCNU1167	Shizhu	2023.3.24	*Quercus* sp.	108.3945°E-29.7076°N (DD)-ca 1060 m	PV156445	PV227300	PV211917	PV175836
***M.diversa* (*Morchella* sp. *Mel*-21)**	FCNU1170	Shizhu	2023.3.24	*Quercus* sp.	108.3945°E-29.7076°N (DD)-ca 1060 m	PV156446	PV227297	PV211915	PV175837
***M.diversa* (*Morchella* sp. *Mel*-21)**	FCNU1180	Qianjiang	2023.3.31	*Pinus* sp.	108.8601°E-29.2948°N (DD)-ca 880 m	PV156363	PV227312	PV211914	PV175842
***M.diversa* (*Morchella* sp. *Mel*-21)**	FCNU1189	Youyang	2023.4.1	Bamboo	109.0195°E-29.2534°N (DD)-ca 800 m	PV156364	PV227295	PV211889	PV175812
***M.diversa* (*Morchella* sp. *Mel*-21)**	FCNU1195	Youyang	2023.4.1	*Quercus* sp.	109.0193°E-29.2526°N (DD)-ca 770 m	PV156365	PV227294	PV211896	PV175813
***M.diversa* (*Morchella* sp. *Mel*-21)**	FCNU1223	Kaizhou	2024.3.26	Unknown	108.5767°E-31.5990°N (DD)-Unknown	PV156377	PV227298	PV211897	PV175814
***M.diversa* (*Morchella* sp. *Mel*-21)**	FCNU1224	Kaizhou	2024.3.26	Unknown	108.5767°E-31.5990°N (DD)-Unknown	PV156378	PV227302	PV211890	PV175815
***M.diversa* (*Morchella* sp. *Mel*-21)**	FCNU1234	Kaizhou	2024.3.26	Unknown	108.5767°E-31.5990°N (DD)-Unknown	PV156379	PV227307	PV211898	PV175816
***M.diversa* (*Morchella* sp. *Mel*-21)**	FCNU1242	Kaizhou	2024.3.26	*Pinus* sp.	108.5737°E-31.6001°N (DD)-ca 1100 m	PV156380	PV227308	PV211918	/
***M.diversa* (*Morchella* sp. *Mel*-21)**	FCNU1247	Kaizhou	2024.3.27	*Pinus* sp.	108.5767°E-31.5990°N (DD)-ca 1020 m	PV156381	PV227299	PV211899	PV175817
***M.diversa* (*Morchella* sp. *Mel*-21)**	FCNU1248	Kaizhou	2024.3.27	*Pinus* sp.	108.5767°E-31.5990°N (DD)-ca 1020 m	PV156382	PV227301	PV211900	PV175818
***M.diversa* (*Morchella* sp. *Mel*-21)**	FCNU1281	Wulong	2024.4.2	*Pinus* sp.	107.8643°E-29.5381°N (DD)-ca 1010 m	PV156383	PV227285	PV211916	PV175819
***M.diversa* (*Morchella* sp. *Mel*-21)**	FCNU1285	Wulong	2024.4.2	*Daphniphyllum* sp.	107.8643°E-29.5381°N (DD)-ca 1010 m	PV156384	PV227309	PV211891	PV175820
***M.diversa* (*Morchella* sp. *Mel*-21)**	FCNU1306	Wulong	2024.4.2	*Pinus* sp.	107.8643°E-29.5381°N (DD)-ca 1010 m	PV156385	/	PV211901	PV175821
***M.diversa* (*Morchella* sp. *Mel*-21)**	FCNU1308	Wulong	2024.4.2	*Pinus* sp.	107.8643°E-29.5381°N (DD)-ca 1010 m	PV156386	PV227303	PV211902	PV175822
***M.diversa* (*Morchella* sp. *Mel*-21)**	FCNU1309	Wulong	2024.4.2	*Daphniphyllum* sp.	107.8643°E-29.5381°N (DD)-ca 1010 m	PV156387	PV227296	PV211903	PV175823
***M.diversa* (*Morchella* sp. *Mel*-21)**	FCNU1310	Wulong	2024.4.2	*Daphniphyllum* sp.	107.8643°E-29.5381°N (DD)-ca 1010 m	PV156388	/	PV211904	PV175824
***M.diversa* (*Morchella* sp. *Mel*-21)**	FCNU1336	Yunyang	2024.4.14	*Pinus* sp.	108.9067°E-30.4672°N (DD)-ca 1320 m	PV156398	PV227310	PV211921	PV175843
***M.diversa* (*Morchella* sp. *Mel*-21)**	FCNU1363	Qianjiang	2024.4.7	Ross	108.7991°E-29.5642°N (DD)-ca 910 m	PV156411	PV227293	PV211924	PV175838
***M.diversa* (*Morchella* sp. *Mel*-21)**	FCNU1412	Nanchuan	2024.3.7	*Pinus* sp.	107.3467°E-29.0314°N (DD)-ca 1000 m	PV156421	PV227286	PV211919	PV175825
***M.diversa* (*Morchella* sp. *Mel*-21)**	FCNU1433	Nanchuan	2024.3.7	*Pinus* sp.	107.3467°E-29.0314°N (DD)-ca 1000 m	PV156422	PV227306	PV211920	PV175826
***M.nipponensis***	FCNU1436	Wuxi	2024.5.10	*Artemisia* sp.	109.7869°E-31.5205°N (DD)-ca 1700 m	PV156423	PV227281	PV227227	PV175884
***M.nipponensis***	FCNU1437	Wuxi	2024.5.10	Weeds	109.7869°E-31.5205°N (DD)-ca 1700 m	PV156424	PV227282	PV227228	PV175885
***M.nipponensis***	FCNU1438	Wuxi	2024.5.10	Weeds	109.7869°E-31.5205°N (DD)-ca 1700 m	PV156425	PV227283	PV227229	PV175886
*M.clivicola*	FCNU1214	Wulong	2023.4.10	*Pyracantha* sp.	108.0037°E-29.2402°N (DD)-ca 1160 m	PV156374	PV227248	PV227211	PV175865
*M.clivicola*	FCNU1355	Wuxi	2024.4.25	Weeds	109.8220°E-31.5080°N (DD)-Unknown	PV156409	PV227269	PV227212	PV175866
*M.clivicola*	FCNU1373	Qianjiang	2024.4.8	*Pinus* sp.	108.6225°E-29.1366°N (DD)-ca 1150 m	PV156417	PV227275	PV227213	PV175867
*M.esculenta*	FCNU1348	Wuxi	2024.4.14	Weeds	109.7873°E-31.5301°N (DD)-ca 1830 m	PV156407	PV227279	PV227226	PV175871
*M.galilaea*	FCNU1443	Shapingba	2020.10.25	*Hibiscus* sp.	106.2983°E-29.6155°N (DD)-ca 290 m	PV156429	PV227268	PV227210	PV175874
*M.sextelata*	FCNU1340	Wuxi	2024.4.21	Unknown	Unknown-ca 1690 m	PV156402	PV227313	PV211911	PV175844
*M.sextelata*	FCNU1342	Wuxi	2024.4.21	Unknown	Unknown-ca 1690 m	PV156403	PV227314	PV211912	PV175845
*M.sextelata*	FCNU1344	Wuxi	2024.4.23	*Pinus* sp.	109.7858°E-31.4687°N (DD)-ca 1660 m	PV156404	PV227315	PV211913	PV175846
*M.pulchella*	FCNU1327	Wuxi	2024.3.30	*Pinus* sp.	109.8775°E-31.4743°N (DD)-ca 1310 m	PV156393	/	PV211909	PV175839
*M.pulchella*	FCNU1346	Wuxi	2024.4.8	*Pinus* sp.	109.8775°E-31.4743°N (DD)-ca 1310 m	PV156405	/	PV211922	PV175841
*M.pulchella*	FCNU1347	Wuxi	2024.4.8	*Pinus* sp.	109.8775°E-31.4743°N (DD)-ca 1310 m	PV156406	PV227311	PV211923	PV175840
*Morchella* sp. Mes-10	FCNU1442	Wuxi	2024.5.13	Weeds	109.8740°E-31.5123°N (DD)-ca 2220 m	PV156428	PV227272	PV227220	PV175883
*Morchella* sp. Mes-12	FCNU1198	Pengshui	2023.4.9	*Corydalis* sp.	108.0493°E-29.2233°N (DD)-ca 990 m	PV156366	PV227242	PV227188	PV175847
*Morchella* sp. Mes-12	FCNU1203	Pengshui	2023.4.9	*Ficus* sp.	108.0795°E-29.2365°N (DD)-ca 1010 m	PV156369	PV227245	PV227189	PV175848
*Morchella* sp. Mes-12	FCNU1205	Pengshui	2023.3.9	*Ficus* sp.	108.0795°E-29.2365°N (DD)-ca 1010 m	PV156370	PV227249	PV227190	PV175849
*Morchella* sp. Mes-12	FCNU1338	Yunyang	2024.4.14	*Pinus* sp.	108.9067°E-30.4672°N (DD)-ca 1320 m	PV156400	PV227250	PV227191	PV175856
*Morchella* sp. Mes-12	FCNU1339	Yunyang	2024.4.14	*Pinus* sp.	108.9067°E-30.4672°N (DD)-ca 1320 m	PV156401	PV227251	PV227192	PV175853
*Morchella* sp. Mes-12	FCNU1376	Qianjiang	2024.4.8	*Pinus* sp.	108.6225°E-29.1366°N (DD)-ca 1150 m	PV156418	PV227243	PV227194	PV175854
*Morchella* sp. Mes-12	FCNU1379	Qianjiang	2024.4.8	*Pinus* sp.	108.6225°E-29.1366°N (DD)-ca 1150 m	PV156419	PV227244	PV227195	PV175855
*Morchella* sp. Mes-23	FCNU1211	Wulong	2023.4.10	*Pyracantha* sp.	108.0047°E-29.2378°N (DD)-ca 1120 m	PV156373	PV227246	PV227197	PV175850
*Morchella* sp. Mes-23	FCNU1217	Pengshui	2023.4.11	*Setaria* sp.	107.9998°E-29.2103°N (DD)-ca 1270 m	PV156376	PV227247	PV227196	PV175851
*Morchella* sp. Mes-25	FCNU1440	Wuxi	2024.5.13	Unknown	109.8732°E-31.5119°N (DD)-ca 2150 m	PV156426	PV227267	PV227218	PV175881
*Morchella* sp. Mes-25	FCNU1441	Wuxi	2024.5.13	Unknown	109.8734°E-31.5119°N (DD)-ca 2060 m	PV156427	PV227280	PV227219	PV175882

^*^: only the three samples marked with “^*^” collected from Shaanxi, China. All the other samples collected from Chongqing, China.

To maximize coverage of *Morchella* species diversity in Chongqing, we implemented an extensive spatial sampling strategy. Given that the local fruiting period of *Morchella* is extremely short—typically about one month, and often shorter owing to rapid temperature increases—it is less applicable to perform repeated temporal sampling at individual sites. Accordingly, during each fruiting season, we prioritized surveying as many different localities as possible rather than revisiting the same sites in successive years. This strategy was intended to yield a more comprehensive view of species diversity and distribution patterns of *Morchella* in Chongqing.

### ﻿Specimens used for sequencing in this study

Two to three samples from each collection site—326 samples in total—were chosen for molecular analysis after preliminary macroscopic observation of the fruiting bodies (Figs 1–3). As a result, 329 ribosomal internal transcribed spacer (ITS) rDNA sequences were generated from the 326 selected samples, along with three samples from Shaanxi, China, relevant to this study. Based on the initial phylogenetic analyses of the ITS dataset, a total of 84 samples—48 from the Esculenta clade (yellow morels) and 36 from the Elata clade (black morels)—were selected for further multi-gene phylogenetic study. The selection criteria specified one or two samples from each district or county within each lineage identified in the ITS dataset, ensuring that the genetic diversity within the 329-specimen dataset was well represented. Detailed information on the final 84 samples is provided in Table 1. To rigorously assess species diversity within these 84 collections, additional PCR amplification and sequencing were performed for portions of three protein-coding genes: the translation elongation factor 1-alpha (*EF1-α*), the RNA polymerase largest subunit (*RPB1*), and the RNA polymerase second largest subunit (*RPB2*).

### ﻿DNA extraction, PCR amplification, DNA sequencing, and phylogenetic analyses

Genomic DNA was extracted from silica gel-dried specimens using the cetyltrimethylammonium bromide (CTAB) method (Doyle and Doyle 1987). A total of four nuclear loci were selected: ITS, *EF1-α*, *RPB1*, and *RPB2*. The primers used for PCR amplification and sequencing of the four genes are listed in Table 2. Each PCR reaction contained 22 µL of T3 Super PCR Mix (Beijing Tsingke Biotech Co., Ltd., Beijing, China), 1 µL of each primer (Sangon Co., Ltd., Shanghai, China), and 1 µL of template DNA; the final volume was 25 µL. PCRs were conducted in a T1000 Thermal Cycler (Bio-Rad, Singapore) using the cycling parameters shown in Table 3. Amplicons were electrophoresed in 1.5% agarose (Sangon Co., Ltd., Shanghai, China) in 1× TAE, stained with GoldView™ (Chongqing Siding Biotech Ltd., Chongqing, China), and then photographed under an ultraviolet transilluminator (Beijing Labgic Technology Co., Ltd., Beijing, China). PCR products were sequenced using an ABI 3730 capillary sequencer (Sangon Co., Ltd., Shanghai, China). Newly generated sequences were assembled and edited using SeqMan v.7.1.0 (DNASTAR package; DNAStar Inc., Madison, WI, United States). Sequence alignments were performed separately for each locus with MAFFT v6.853 using the E-INS-i strategy (Katoh et al. 2002), manually checked with BioEdit v.7.0.9 (Hall 1999), and concatenated using SequenceMatrix v.1.7.8 (Vaidya et al. 2011).

**Table 2.** Details of primers used for PCR amplification in this study.

Locus	Primer	Sequence (5’-3’)	Reference
ITS	ITS1	TCCGTAGGTGAACCTGCGG	(White et al. 1990)
	ITS4	TCCTCCGCTTATTGATATGC	
	ITS5	GGAAGTAAAAGTCGTAACAAGG	
	ITS1F	CTTGGTCATTTAGAGGAAGTAA	(Gardes and Bruns 1993)
*EF1-a*	EF-1R	GGARGGAAYCATCTTGACGA	(Du et al. 2012b)
	EF-1L	TCTCGCSTACAYCTTGGTG	
	EF-2218R	ATGACACCRACRGCRACRGTYTG	(Rehner and Buckley 2005)
	EF-595F	CGTGACTTCATCAAGAACATG	(Kauserud and Schumacher 2001)
*RPB1*	RPB1B-R	GCCTCRAATTCGTTGACRACGT	(Du et al. 2012b)
	RPB1B-F	AACCGGTATATCACGTYGGTAT	
	RPB1Y-R	GTTGACAACGTGAGCTGGAGA	
	RPB1Y-F	CGATCTATTAGAACATGGGGCTTC	
	RPBC2	GMAGAACMGTAATCACCATCC	(Taşkın et al. 2010)
	RPBA2	GTTAGATGAAGTGAGACACAC	
*RPB2*	RPB2B-R	GATACCATGGCGAACATTCTG	(Du et al. 2012b)
	RPB2B-F	TAGGTAGGTCCCAAGAACACC	
	RPB2Y-R	CACGGCTCTGGTATCCATTC	
	RPB2Y-F	CTTGCCACTACGCGGTCTAT	
	RPB2-3R	GCATYGGTATGCAGGTTGTGG	(Taşkın et al. 2010)
	RPB2-9F	CAAATGGGCRATTGTCATACG	

**Table 3.** PCR programs used for amplification of ITS, *EF1-a*, *RPB1*, and *RPB2* in this study.

Gene	PCR Program
ITS	2’ −98 °C, 35× (10” −98 °C, 10” −50 °C , 20” −72 °C), 10’ −72 °C
*EF1-a*	2’ −98 °C, 35× (10” −98 °C, 10” −50 °C, 90” −72 °C), 10’ −72 °C
*RPB1*	2’ −98 °C, 35× (10” −98 °C, 10” −50 °C, 90” −72 °C), 10’ −72 °C
*RPB2*	2’ −98 °C, 35× (10” −98 °C, 10” −50 °C, 90” −72 °C), 10’ −72 °C

Three datasets were then constructed: a single ITS dataset of *Morchella* (329 sequences); a combined four-gene (ITS, EF1-α, RPB1, and RPB2) dataset for the Esculenta clade (48 sequences, 13 species); and a combined four-gene dataset for the Elata clade (36 sequences, 3 species). These sequences were integrated with previously published multilocus DNA sequences of *Morchella* species retrieved from GenBank (O’Donnell et al. 2011; Du et al. 2012a, 2019; Elliott et al. 2014; Pildain et al. 2014; Voitk et al. 2014; Loizides et al. 2016; Baroni et al. 2018; Machuca et al. 2021; Clowez et al. 2022; Cravero et al. 2024) and analyzed under the criteria of GCPSR to examine the phylogenetic relationships among the newly collected samples and previously published *Morchella* species. *Morchellarufobrunnea* was used as the outgroup for the ITS dataset (330 sequences), and both *M.rufobrunnea* and *M.anatolica* were chosen as the outgroups for the final four-gene datasets of the Esculenta clade (115 sequences, 39 species) and the Elata clade (117 sequences, 46 species). The corresponding GenBank accession numbers for the retrieved sequences of representative species in the Elata and Esculenta clades of *Morchella* and their reference sources are provided in Suppl. material 1: tables S1, S2, respectively.

Maximum likelihood (ML) phylogenetic analysis was performed for the ITS dataset using RAxML v.8.2.12 (Stamatakis 2014) for initial assessment. Both ML and Bayesian inference (BI) phylogenetic analyses were conducted for the two combined four-gene datasets using RAxML v.8.2.12 (Stamatakis 2014) and MrBayes v.3.2.7a (Ronquist et al. 2012), respectively. A rapid bootstrapping with 1,000 replicates was executed for the ML analysis with the GTR+GAMMA+I model selected by ModelTest v.3.8 (Posada 2006). The BI analysis was run for one million generations, sampling trees every 100 generations, using four Markov Chain Monte Carlo (MCMC) chains. The runs were terminated when the mean standard deviation of split frequencies fell below 0.01. The top 25% of sampled trees were discarded as burn-in using the “sumt” and “sump” commands to obtain posterior probabilities. Phylogenetic trees were visualized and annotated in FigTree v.1.4.4 (Rambaut 2008).

### ﻿Morphological observation

Macro-morphological characteristics were recorded based on field notes and photographs of fresh ascomata. Micro-morphological descriptions of new species within *Morchella* reported in this study were conducted using a microscope. This included observations of asci (the fertile elements of the hymenium), paraphyses (the sterile elements of the hymenium), acroparaphyses (the sterile elements of the ridges), and ascospores. Additionally, the number of ascospores per ascus was recorded. Measurements of ascospores were made under 95% confidence intervals, with sizes presented using range notation in the format (a–) b–c (–d). Here, the range b–c represents at least 90% of the measured values, while extreme values (a, d) are included in parentheses. *Q* refers to the length/width ratio of an individual ascospore, and *Qm* refers to the average *Q* of all ascospores ± sample standard deviation.

Hand sections for microscopic examination were prepared using a safety razor blade. Specimens were stained with 1% aqueous Congo Red solution prior to morphological analysis. Observations of microscopic features were conducted using an Optec BK-FL light microscope (Optec, Chongqing, China) at magnifications of 20×, 40×, and 100×. Images were captured with an Optec CCD TP510 digital camera and processed using Adobe Photoshop CC 2019 (v.20.0.4). For scanning electron microscopy (SEM) analysis, fragments scraped from the hymenium were mounted on aluminum stubs with double-sided adhesive tape, coated with a gold-palladium alloy, and examined using a Hitachi SU3500 scanning electron microscope (Hitachi, Japan).

### ﻿Local temperature acquisition

**Figure 4.**
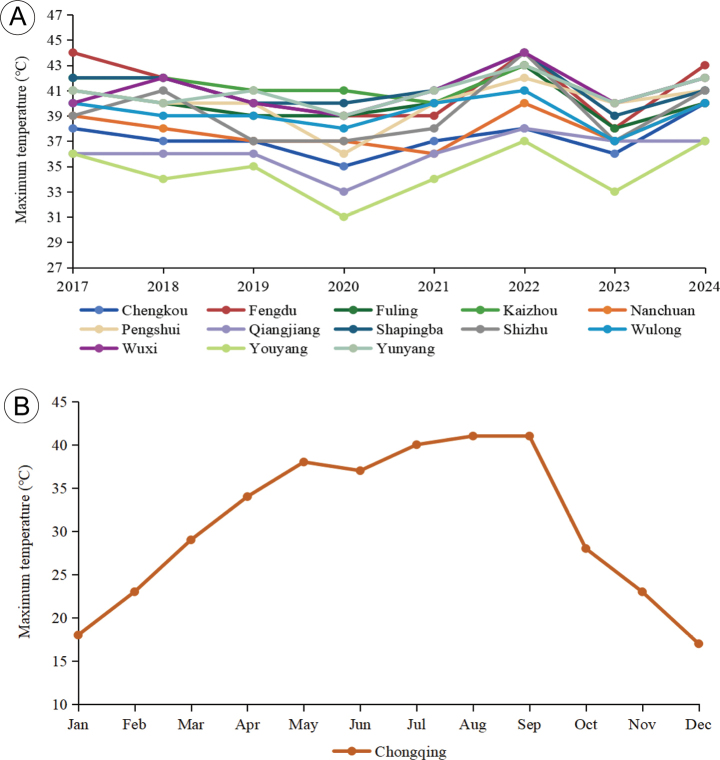
Maximum temperatures recorded from 2017 to 2024 in Chongqing. A. Annual maximum temperatures from 2017 to 2024 in 13 districts and counties in Chongqing. B. Monthly maximum temperatures in 2024 in Chongqing.

Temperature data were acquired from the local meteorological stations of 13 districts and counties in Chongqing investigated in this study. Annual maximum temperature records from 2017 to 2024 for each district and county were extracted and used to assess whether local temperatures affected species diversity (Fig. 4A; Suppl. material 1: table S3). In addition, taking 2024 as a representative year, the municipality’s monthly maximum temperatures were analyzed to characterize its seasonal thermal regime (Fig. 4B; Suppl. material 1: table S4).

## ﻿Results

### ﻿Phylogenetic analysis of the ITS dataset

**Figure 5.**
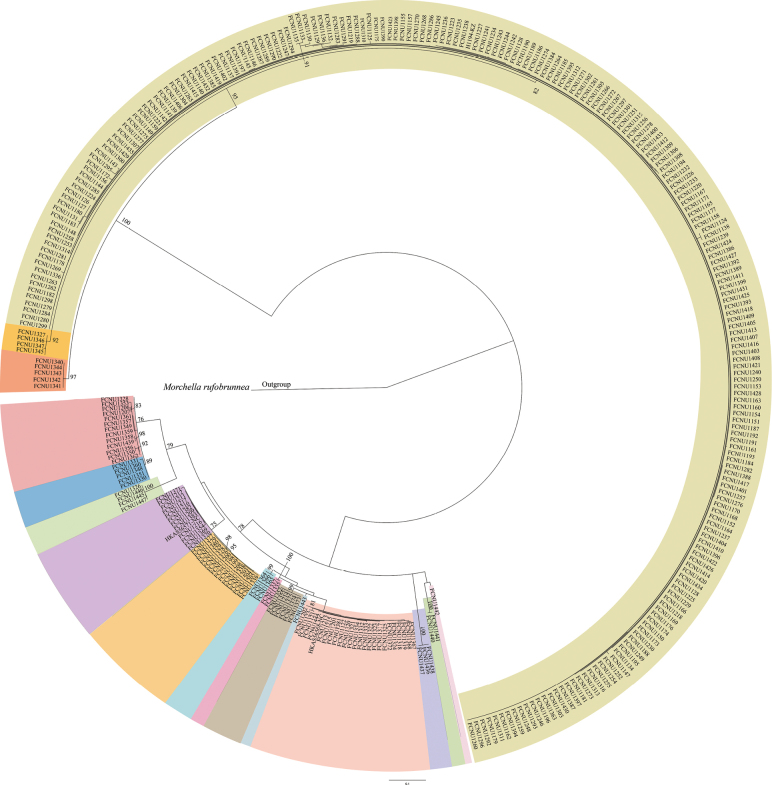
The phylogenetic tree of 17 species of *Morchella*. It’s inferred from ML analyses based on the ITS dataset generated from 330 sequences. Bootstrap values over 75% indicated on the branches. The different-colored branches represent the different species in *Morchella*. *Morchellarufobrunnea* was chosen as the outgroup.

During an eight-year survey conducted in Chongqing, China, 326 representative samples were selected for ITS sequencing to perform phylogenetic analysis. These samples were preliminarily screened from all collected morel specimens based on their macro-morphologies and geographical localities. The final ITS dataset comprised 330 sequences (326 from Chongqing, three from Shaanxi, and one outgroup) with an aligned length of 1,408 bp, providing a preliminary assessment of *Morchella* species diversity in Chongqing. Maximum likelihood phylogenetic analysis of the ITS dataset revealed 16 potential *Morchella* species from Chongqing samples, grouped into two strongly supported clades: the Esculenta clade (yellow morels) and the Elata clade (black morels) (Fig. 5). Subsequently, 48 samples from the Esculenta clade and 36 from the Elata clade—representing the full spectrum of sampled species diversity in Chongqing (Table 1)—were selected for further multi-gene phylogenetic analyses.

### ﻿Phylogenetic analysis of multi-gene datasets of Elata and Esculenta clades

**Figure 6.**
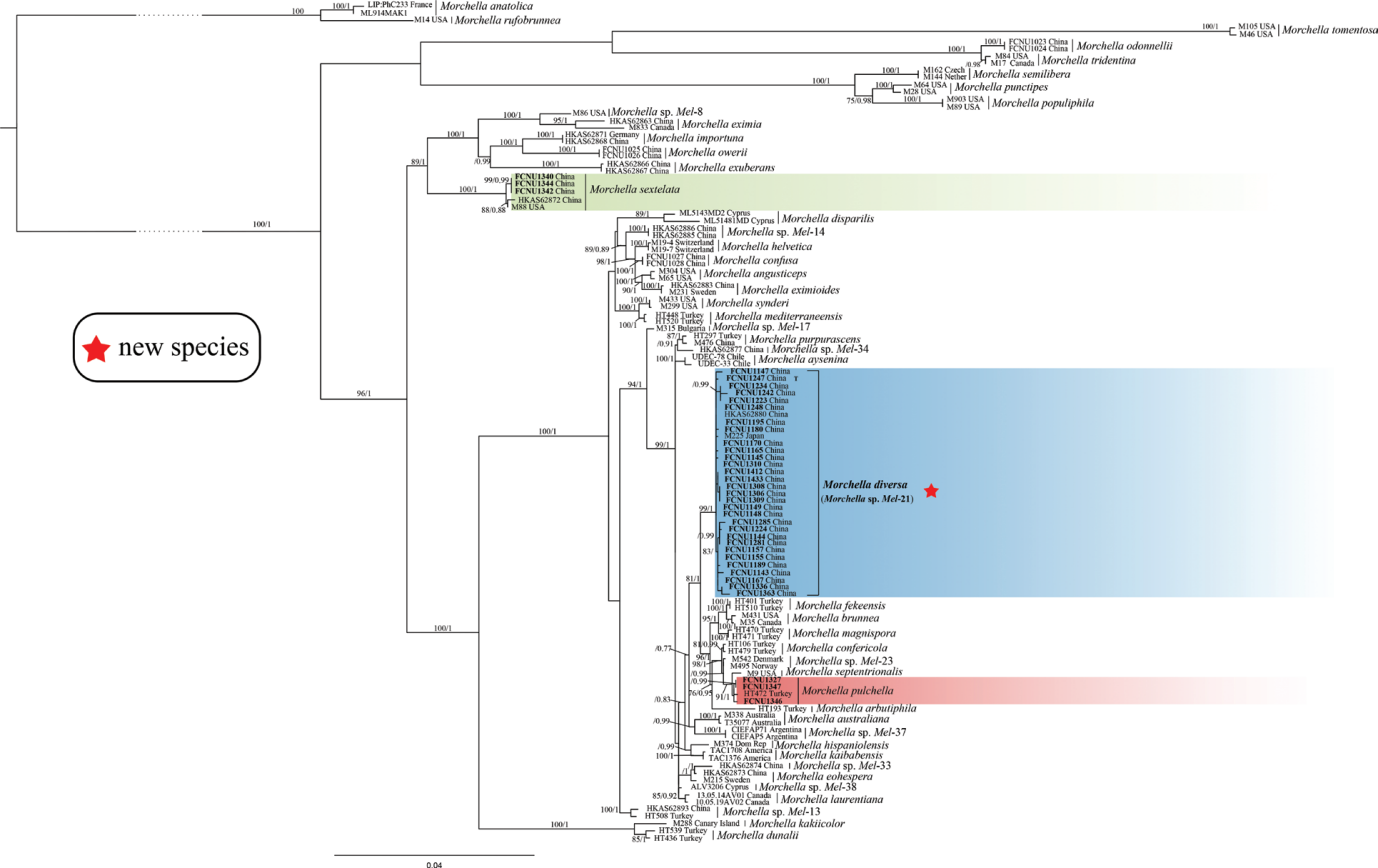
The phylogenetic tree of 36 species in the Elata clade of *Morchella*. It’s inferred from ML analyses based on the concatenated dataset (ITS, *EF1-a*, *RPB1*, and *RPB2*) generated from 117 sequences. Bootstrap values over 75% and Bayesian posterior probabilities over 0.95 are shown on the branches. Three differently colored branches represent the three species identified from Chongqing in the Elata clade in this study, namely green for *M.sextelata*, blue for *M.diversa* (*Morchella* sp. *Mel*-21), and red for *M.pulchella*. Sequences generated from newly collected specimens in this study are indicated in bold. The newly described species is marked with a red star, and its species name is highlighted in bold. The holotype specimen for the new species is indicated by the bold “**T**” following the specimen name.

The four-gene dataset (ITS, *EF1-α*, *RPB1*, and *RPB2*) for the Elata clade totaled 3,667 bp and included 117 sequences, comprising 36 newly collected samples from Chongqing and 81 reference phylospecies retrieved from GenBank (Table 1; Suppl. material 1: table S1). Phylogenetic trees for the Elata clade were constructed using ML and BI analyses. No significant topological differences were observed between the two methods, and the ML tree is presented in Fig. 6.

Within the Elata clade, the studied specimens FCNU1340, FCNU1342, and FCNU1344 from Wuxi County were grouped with *M.sextelata* with high support values (BS = 100%; PP = 1), and specimens FCNU1327, FCNU1346, and FCNU1347, also from Wuxi County, were grouped with *M.pulchella* (BS < 75%, PP < 0.95). In addition, specimens FCNU1143–FCNU1145, FCNU1147–FCNU1149, FCNU1155, FCNU1157, FCNU1165, FCNU1167, FCNU1170, FCNU1180, FCNU1189, FCNU1195, FCNU1223, FCNU1224, FCNU1234, FCNU1242, FCNU1247, FCNU1248, FCNU1281, FCNU1285, FCNU1306, FCNU1308–FCNU1310, FCNU1336, FCNU1363, FCNU1412, and FCNU1433, respectively from Wulong, Kaizhou, Youyang, Shizhu, Qianjiang, Yunyang, Pengshui, and Nanchuan, were grouped with *Mel*-21 (BS = 99%; PP = 1) (Fig. 6). Thus, three species from the Elata clade were identified from Chongqing, namely *M.sextelata*, *M.pulchella*, and *Morchella* sp. *Mel*-21 (Figs 1–3, 6).

**Figure 7.**
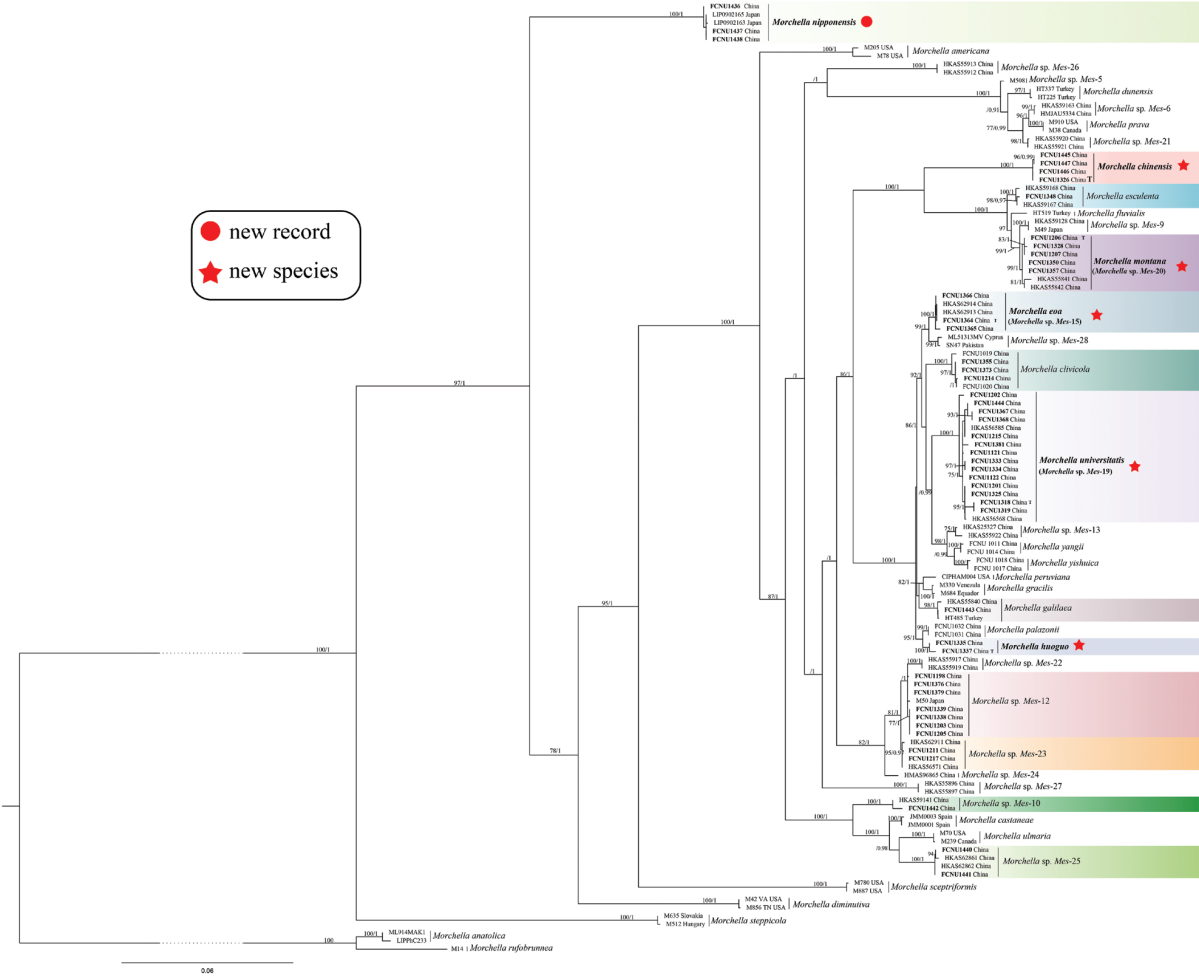
The phylogenetic tree of 48 species in the Esculenta clade of *Morchella*. It’s inferred from ML analyses based on the concatenated dataset (ITS, *EF1-a*, *RPB1*, and *RPB2*) generated from 115 sequences. Bootstrap values over 75% and Bayesian posterior probabilities over 0.95 are shown on the branches. The differently colored branches correspond to the thirteen species identified from Chongqing in the Esculenta clade of *Morchella*. Sequences generated from newly collected specimens in this study are indicated in bold. The five newly described species are marked with a red star, and the newly recorded species is indicated with a red dot. The species names of the newly described and recorded species are highlighted in bold. The holotype specimens for each new species are indicated by the bold “**T**” following their specimen names.

The four-gene dataset (ITS, *EF1-α*, *RPB1*, and *RPB2*) for the Esculenta clade totaled 3,791 bp and included 115 sequences, comprising 48 newly collected samples from Chongqing and 67 taxa representing reference phylospecies retrieved from GenBank (details provided in Table 1 and Suppl. material 1: table S2). The phylogenetic tree for the Esculenta clade was constructed using ML and BI phylogenetic analyses. No significant topological differences were shown between the two analyses, and the ML phylogenetic tree is displayed in Fig. 7.

Within the Esculenta clade, 32 samples were grouped with nine reference phylospecies, each supported by high values (Fig. 7). Specifically, FCNU1443, from Shapingba, was clustered with *M.galilaea* (BS = 98%, PP = 1), the only species fruiting in autumn in *Morchella*; FCNU1214, FCNU1355, and FCNU1373, from Wulong, Wuxi, and Qianjiang, were grouped with *M.clivicola* (BS = 100%, PP = 1); FCNU1348, from Wuxi, was clustered with *M.esculenta* (BS = 100%, PP = 1); FCNU1442, from Wuxi, was matched with *Mes*-10 (BS = 100%, PP = 1); FCNU1364–FCNU1366, from Qianjiang, were grouped with *Mes*-15 (BS = 100%, PP = 1); FCNU1121, FCNU1122, FCNU1201, FCNU1202, FCNU1215, FCNU1318, FCNU1319, FCNU1325, FCNU1333, FCNU1334, FCNU1367, FCNU1368, FCNU1381, and FCNU1444, from Pengshui, Qianjiang, Wulong, Fuling, Shapingba, Fengdu, Youyang, and Chengkou, were grouped with *Mes*-19 (BS = 100%, PP = 1); FCNU1206, FCNU1207, FCNU1328, FCNU1350, and FCNU1357, from Wuxi and Pengshui, were clustered with *Mes*-20 (BS = 99%, PP = 1); FCNU1211 and FCNU1217, from Wulong and Pengshui, were grouped with *Mes*-23 (BS = 95%, PP = 0.97); and both FCNU1440 and FCNU1441, from Wuxi, were clustered with *Mes*-25 (BS = 100%, PP = 1).

Besides, *M.nipponensis* (BS = 100%, PP = 1; Fig. 7) was discovered for the first time in Chongqing (FCNU1436–FCNU1438 from Wuxi) and represents a new record for China, originally described in Japan. In addition, seven samples (FCNU1198, FCNU1203, FCNU1205, FCNU1338, FCNU1339, FCNU1376, and FCNU1379, from Yunyang, Qianjiang, and Pengshui) were clustered with *Mes*-12; however, they were provisionally identified as putative *Mes*-12 due to insufficient support values (BS < 75%, PP < 0.95).

Notably, FCNU1326 and FCNU1445–FCNU1447 from Chengkou and Shaanxi, as well as FCNU1335 and FCNU1337 from Yunyang, were clustered into two distinct lineages, clearly separated from all other reference species. These lineages represent two phylogenetically distinct new species. Their topological positions were strongly supported by ML bootstrap values (BS = 100%) and BI posterior probabilities (PP = 1) (Fig. 7). Combined with morphological observations, the two newly identified phylospecies were described as *Morchellachinensis* and *Morchellahuoguo* (see section Taxonomy).

Among all the species identified in Chongqing, eight phylospecies were previously reported in other studies, namely *Morchella* sp. *Mel*-21, *Mes*-10, *Mes*-12, *Mes*-15, *Mes*-19, *Mes*-20, *Mes*-23, and *Mes*-25 (Figs 6, 7). Due to insufficient support for *Morchella* sp. *Mel*-21 and a lack of sufficient high-quality specimens for *Mes*-10, *Mes*-12, *Mes*-23, and *Mes*-25, we conducted micro-morphological observations exclusively on *Mes*-15, *Mes*-19, *Mes*-20, and *Mel*-21, which were then described as *Morchellaeoa*, *Morchellauniversitatis*, *Morchellamontana*, and *Morchelladiversa*, respectively (see section Taxonomy).

### ﻿Rich species diversity of *Morchella* identified in Chongqing

Through eight years of extensive sampling in Chongqing, a total of 16 phylospecies were identified, comprising 13 species in the Esculenta clade and three species in the Elata clade (Figs 2, 3, 6, 7). Such rich species diversity in Chongqing had been previously unexpected, and the significant disparity in species numbers between the two clades is particularly noteworthy. The Esculenta clade not only exhibits considerable species diversity but also includes two newly identified species and one species recorded for the first time in China. In contrast, the Elata clade is represented by only three species, underscoring the stark difference in diversity compared to the Esculenta clade. The results indicate that ecological and environmental factors in Chongqing likely contribute to the different species diversity observed between the Elata and Esculenta clades in *Morchella*.

### ﻿Divergent distribution pattern of *Morchella* species uncovered in Chongqing

The investigation of *Morchella* species in Chongqing has unveiled a pronounced divergence in the distribution patterns of the Elata and Esculenta clades. Within the Elata clade, the three recognized species—*M.sextelata*, *M.pulchella*, and *M.diversa* (*Morchella* sp. *Mel*-21)—exhibit contrasting distribution patterns in Chongqing (Figs 1, 3). *Morchellasextelata* and *M.pulchella* were exclusively discovered in Wuxi County, while *M.diversa* (*Morchella* sp. *Mel*-21) demonstrates a considerably broader distribution, occurring across eight districts and counties, thereby serving as the most widespread species in the Elata clade in Chongqing. Furthermore, *M.diversa* (*Morchella* sp. *Mel*-21) is the sole *Morchella* species discovered in both Kaizhou District and Shizhu County. However, no species of the Elata clade were identified in the four explored regions: Shapingba, Fengdu, Fuling, and Chengkou.

Within the Esculenta clade, *M.universitatis* (*Morchella* sp. Mes-19) is recognized as the most widely distributed species in Chongqing, having been discovered in nine districts and counties (Figs 1, 2). Moreover, *M.universitatis* (*Morchella* sp. Mes-19) is the sole *Morchella* species discovered in Fuling and Fengdu. In contrast, *M.galilaea* displays a highly restricted distribution, being exclusively found in Shapingba (Fig. 1). Similarly, *M.eoa* (*Morchella* sp. Mes-15) is confined to Qianjiang District, while *M.esculenta*, *M.nipponensis*, *Morchella* sp. Mes-10, and *Morchella* sp. Mes-25 are limited to Wuxi County (Fig. 1).

The distribution of *Morchella* species in Chongqing shows an uneven pattern (Fig. 1). Among the 13 districts and counties investigated, Wuxi emerges as a center and hotspot for *Morchella* species diversity in Chongqing, with a total of eight species identified (Fig. 1). This includes six species from the Esculenta clade and two from the Elata clade. Notably, six of these species have only been found in Wuxi, specifically *M.nipponensis*, *M.esculenta*, *M.sextelata*, *M.pulchella*, *Mes*-10, and *Mes*-25. Qianjiang District and Pengshui County serve as the second and third species diversity centers of *Morchella* in Chongqing, respectively. This indicates that the southeastern and northeastern parts of Chongqing are the primary distribution centers for *Morchella* species, with a limited distribution in the central and western regions.

In general, *M.diversa* (*Morchella* sp. *Mel*-21) and *M.universitatis* (*Morchella* sp. Mes-19) are recognized as the most widespread species in the Elata and Esculenta clades, respectively, in Chongqing. Both species exhibit a pronounced concentration in the southeastern and northeastern regions of Chongqing, particularly within the Daba and Wuling Mountains. This spatial preference suggests that the mountainous environment and ecological conditions likely play a critical role in shaping the distribution patterns of the two species.

### ﻿Taxonomy

#### ﻿ Morchella chinensis

Taxon classificationAnimaliaPezizalesMorchellaceae

Q. Qin & X.H. Du sp. nov.

7EB6A88D-C1CD-546A-A752-36BCCB61DA3D

857691

Figs 2A, B , 8A, B

##### Etymology.

The specific epithet chinensis (Latin, chinensis = “of China”) refers to the country of origin of the type specimen, China.

**Figure 8.**
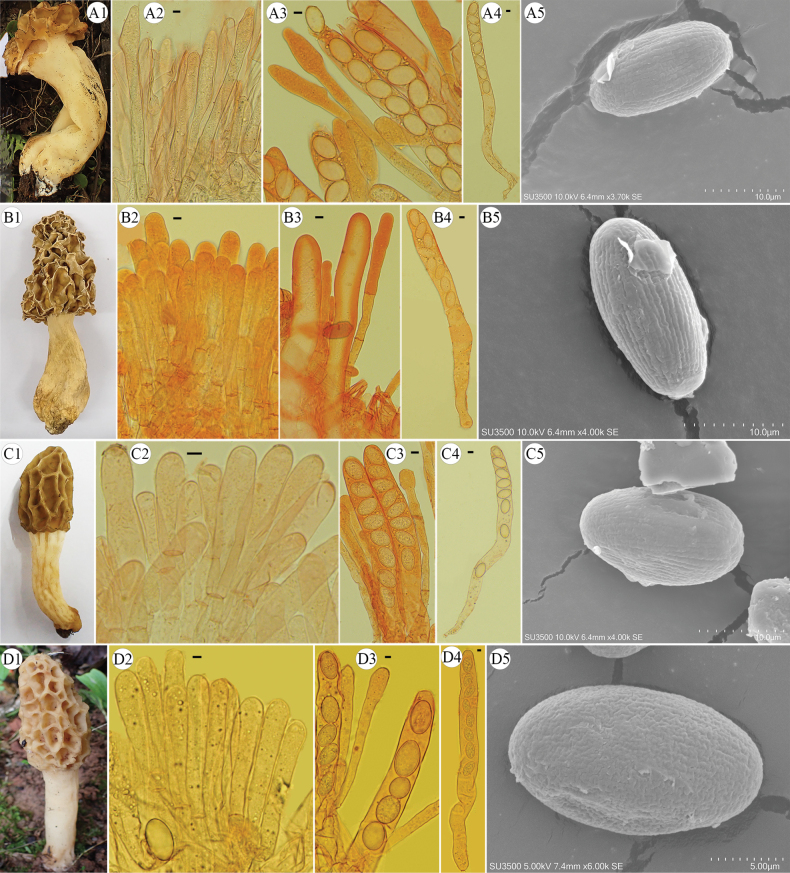
Morphological characteristics of new species, *Morchellachinensis, Morchella huguo*, and *Morchellaeoa*, discovered in this study. A, B. Macro- and micro-morphological characters of *Morchellachinensis* sp. nov. (A. FCNU1326; B. FCNU1446). A1, B1. Ascocarps. A2, B2. Acroparaphyses; A3, B3. Paraphyses. A4, B4. Asci. A5, B5. SEM images of ascospores, with regular longitudinal ornamentation. C. Macro- and micro-morphological characters of *Morchellahuoguo* sp. nov. (FCNU1337). C1. Ascocarp. C2. Acroparaphyses. C3. Paraphyses. C4. Ascus. C5. SEM image of an ascospore, with light irregular longitudinal and interconnecting transverse ornamentation. D. Macro- and micro-morphological characters of *Morchellaeoa* sp. nov. (FCNU1364). D1. Ascocarp. D2. Acroparaphyses. D3. Paraphyses. D4. Ascus. D5. SEM image of an ascospore, with irregular longitudinal and interconnecting transverse ornamentation. Scale bars: 5 μm (A2–A4, B2–B4, C2–C4, D2–D5); 10 μm (A5, B5, C5).

##### Diagnosis.

Late-appearing, medium- to large-sized morel, characterized by an ovoid or bluntly conical pileus with deep and large pits in irregular sizes and with primary vertical and transecting horizontal ridges. Stipe long, usually inflated at the base, glabrous or finely mealy with yellowish granules. Paraphyses slender, obviously inflated at the apex. Usually occurring in subtropical broad-leaved forests, associated with *Quercus* sp., *Juglans* sp., and *Castanea* sp., and sometimes beside weeds.

##### Type.

**China • 1**; Chongqing Municipality, Chengkou County. 26 Apr 2024, Q. Qin (FCNU1326—***holotype*** preserved in the Fungal Herbarium of Chongqing Normal University). GenBank: ITS = PV156392; *EF1-a* = PV227241; *RPB1* = PV227235; *RPB2* = PV204696.

##### Description.

Ascomata 91 mm high. Hymenophore 17 mm high; 43 mm wide at the widest point; ovoid with a convex or bluntly conical; the hymenophore attached to the stipe with a sinus, 10–12 primary vertical ridges and some shorter, secondary vertical ridges, and transecting horizontal ridges, forming a honeycomb-like structure. Ridges finely tomentose; pale yellow to dark brown. Pits asymmetrical; deep and irregular in outline and size; glabrous or finely tomentose; primarily elongated vertically; pale yellowish to yellowish brown. Stipe clavate to subclavate, 74 mm high; 23 mm wide; glabrous or finely mealy with yellowish granules. Context whitish; 2–3 mm thick in the hollow hymenophore; sometimes slightly chambered near the base. Sterile inner surface whitish and pubescent.

Ascospores measuring (19.36-) 19.98–25.08 (-25.78) × (12.37-) 12.49–14.88 (-15.79) μm (Q = 1.46–1.78, Qm = 1.65 ± 0.08), elliptical to ovoid, with obviously regular longitudinal ornamentation; contents homogeneous. Asci cylindrical, eight-spored, (299.92-) 310.98–385.80 (-388.74) × (18.26-) 19.85–24.54 (-26.92) μm. Paraphyses, cylindrical in shape, apices generally rounded but occasionally subclavate to clavate, septate, (123.98-) 130.51–239.75 (-263.98) × (10.35-) 10.73–17.78 (-23.32) μm, but difficult to measure their length. Elements on sterile ridges (105.09-) 116.22–214.51 (-226.41) × (10.96-) 13.57–19.30 (-21.07) μm, difficult to measure their length. The number of ascospores in asci observed to vary among four, six, seven, and eight.

##### Habitat.

Solitary on the ground nearby weeds.

##### Distribution.

This species is only known from Chongqing, China, and Shaanxi, China.

##### Other specimens examined.

**China • 1**; Shaanxi Province, Ankang City, 15 May 2017, Z.D. Yu (FCNU1445—preserved in the Fungal Herbarium of Chongqing Normal University). **China • 1**; Shaanxi Province, Ankang City, 15 May 2017, Z.D. Yu (FCNU1446—preserved in the Fungal Herbarium of Chongqing Normal University). **China • 1**; Shaanxi Province, Xi’an City, Heihe Forest Park, May 2017, Z.D. Yu (FCNU1447—preserved in the Fungal Herbarium of Chongqing Normal University).

##### Comments.

In comparison to *M.esculenta* and several other species clustered as the sister species, the ascocarp of *M.chinensis* is usually more sharply conical than them, and its stipe is proportionally longer. In addition, the distribution of *M.chinensis*, currently only known from Shaanxi and Chongqing, China, is more limited than *M.esculenta*, which is widely distributed in Europe and Asia.

#### ﻿ Morchella huoguo

Taxon classificationAnimaliaPezizalesMorchellaceae

Q. Qin & X.H. Du sp. nov.

99C021C2-1350-5C9B-99DC-FE05FC3B69D9

857692

Figs 2C , 8C

##### Etymology.

The specific epithet huoguo (火锅, Mandarin Pinyin: huǒguō) refers to hotpot, a globally renowned culinary tradition originating from the type locality of this species, Chongqing, China. The name honors Chongqing’s iconic gastronomic cultural heritage while phonetically preserving the Mandarin term for “hotpot” in Latinized form.

##### Diagnosis.

Middle-appearing, small- to middle-sized morel, characterized by a bluntly conical pileus with very large, deep, and relatively sparse pits in irregular size and with primary vertical and few transecting horizontal ridges. Stipe long, usually slightly inflated at the top, obviously wrinkled at the surface, glabrous or finely mealy with yellowish granules. Paraphyses slender, inflated at the apex. Occurring under *Pinus* sp.

##### Type.

**China • 1**; Chongqing Municipality, Yunyang County. 14 Apr 2024, Q. Qin (FCNU1337—***holotype*** preserved in the Fungal Herbarium of Chongqing Normal University). GenBank: ITS = PV156399; *EF1-a* = PV227237; *RPB1* = PV227231; *RPB2* = PV204692.

##### Description.

Ascomata 79 mm high. Hymenophore 37 mm high; 23 mm wide at the widest point; conical or bluntly conical; the hymenophore attached to the stipe with a notable sinus; pitted and ridged, with 12–15 primary vertical ridges and some shorter, secondary vertical ridges, and transecting horizontal ridges, forming a honeycomb-like structure. Ridges slender, finely tomentose; pale yellowish to yellow-brown. Pits asymmetrical, mainly large; primarily elongated vertically; deep and irregular in outline and size; glabrous or finely tomentose; yellowish. Stipe cylindrical, 42 mm high; 12 mm wide; glabrous or finely mealy with whitish granules; whitish to pale yellowish. Context whitish; 1–2 mm thick in the hollow hymenophore; sometimes slightly chambered near the base. Sterile inner surface whitish and pubescent.

Ascospores measuring (17.78-) 19.87–23.78 (-24.66) × (11.47-) 11.83–15.22 (-15.99) μm (Q = 1.34–1.85, Qm = 1.60 ± 0.14), elliptical to ovoid, with light irregular longitudinal and interconnecting transverse ornamentation; contents homogeneous. Asci cylindrical, eight-spored, (158.68-) 180.73–356.59 (-359.35) × (10.93-) 11.45–29.92 (-30.10) μm. Paraphyses, cylindrical in shape, apices generally rounded but occasionally subclavate to clavate, septate, (105.59-) 115.34–169.05 (-171.49) × (7.60-) 9.86–14.84 (-16.94) μm, but difficult to measure their length. Elements on sterile ridges (85.83-) 88.14–172.47 (-199.01) × (9.19-) 9.36–17.57 (-17.92) μm, difficult to measure their length. The number of ascospores in asci observed to vary from one to eight.

##### Habitat.

Solitary on the ground under *Pinus* sp.

##### Distribution.

This species is only recorded from Chongqing, China.

##### Other specimens examined.

**China • 1**; Chongqing Municipality, Yunyang County. 14 Apr 2024, Q. Qin (FCNU1335—***paratype*** preserved in the Fungal Herbarium of Chongqing Normal University).

##### Comments.

*Morchellahuoguo* is phylogenetically close to *M.palazonii*, making them sister species. However, they are easy to distinguish by morphological features, particularly pit size and density, with *M.huoguo* having fewer and larger pits.

#### ﻿ Morchella eoa

Taxon classificationAnimaliaPezizalesMorchellaceae

Q. Qin & X.H. Du sp. nov.

4F4168F2-2931-5068-A6F5-0A69D9426C50

859399

Figs 2H–J , 8D

##### Etymology.

The specific epithet eoa is derived from the Latinized Greek word eōas (from Eōs, the goddess of dawn), meaning “eastern” or “of the dawn.” It refers to the species’ distribution currently only found in Eastern Asia.

##### Diagnosis.

Middle-appearing, small- to middle-sized morel, characterized by an oval or bluntly conical pileus with middle, deep, and relatively dense pits in irregular and polygonal sizes and primary vertical and rich transecting horizontal ridges. Stipe whitish to yellowish, of medium length, with a distinctly wrinkled surface, glabrous or finely mealy with yellowish granules, without obvious inflated. Paraphyses slender, slightly inflated at the apex. Occurring under *Pinus* sp.

##### Type.

**China • 1**; Chongqing Municipality, Qianjiang District. 7 Apr 2024, Q. Qin (FCNU1364—***holotype*** preserved in the Fungal Herbarium of Chongqing Normal University). GenBank: ITS = PV156412; *EF1-a* = PV227270; *RPB1* = PV227209; *RPB2* = PV175872.

##### Description.

Ascomata 124 mm high. Hymenophore 62 mm high; 34 mm wide at the widest point; conical to bluntly conical; pale brownish yellow or yellowish; with 9–11 primary vertical ridges and some shorter, secondary vertical ridges, and transecting horizontal ridges, forming a honeycomb-like or reticulate pattern. Ridges undulate, forming maze-like irregular geometric patterns; light yellowish-brown. Pits irregular polygonal, with serrated margins, moderate light brown. Stipe clavate to subclavate, 62 mm high; 16 mm wide; glabrous or finely mealy with yellowish granules. Context whitish; 2–3 mm thick in the hollow hymenophore; sometimes slightly chambered near the base. Sterile inner surface whitish and pubescent.

Ascospores (16.93-) 18.40–23.00 (-28.12) × (7.93-) 10.87–15.24 (-15.62) μm (Q = 1.37–3.55, Qm = 1.60 ± 0.34), elliptical to ovoid, with nearly irregular longitudinal and interconnecting transverse ornamentation; contents homogeneous. Asci cylindrical, eight-spored, (236.10-) 251.78–254.87 (-371.77) × (17.58-) 19.20–24.88 (-25.19) μm. Paraphyses, cylindrical, septate, apices generally merely rounded but occasionally subclavate, (113.03-) 115.07–174.92 (-177.91) × (10.84-) 10.90–16.49 (-19.65) μm, but difficult to measure their length. Elements on sterile ridges (75.37-) 80.45–131.94 (-150.68) × (12.05-) 12.11–20.32 (-21.02) μm. The number of ascospores in asci observed to vary from one to nine.

##### Habitat.

Solitary on the ground under *Pyrus* sp. and *Populus* sp.

##### Distribution.

Currently, this species is only recorded from southwestern China, such as Chongqing, Xizang, and Yunnan.

##### Other specimens examined.

**China • 1**; Chongqing Municipality, Qianjiang District. 7 Apr 2024, Q. Qin (FCNU1365—***paratype*** preserved in the Fungal Herbarium of Chongqing Normal University). **China • 1**; Chongqing Municipality, Qianjiang District. 7 Apr 2024, Q. Qin (FCNU1366—***paratype*** preserved in the Fungal Herbarium of Chongqing Normal University).

##### Comments.

This morphologically distinct species corresponds to the previously reported phylospecies *Morchella* sp. Mes-15 determined by multi-gene phylogenetic analysis in Du et al. (2012a).

#### ﻿ Morchella universitatis

Taxon classificationAnimaliaPezizalesMorchellaceae

Q. Qin & X.H. Du sp. nov.

5D7966F5-4504-5410-8B91-9C15C136A58B

859401

Figs 2M–R , 9A

##### Etymology.

The specific epithet universitatis comes from the Latin noun universitas, -atis (f.), meaning “university” or “community of scholars.” Used here in the genitive singular, universitatis translates as “of the university,” referring to the species’ recurrent occurrence on university campuses.

**Figure 9.**
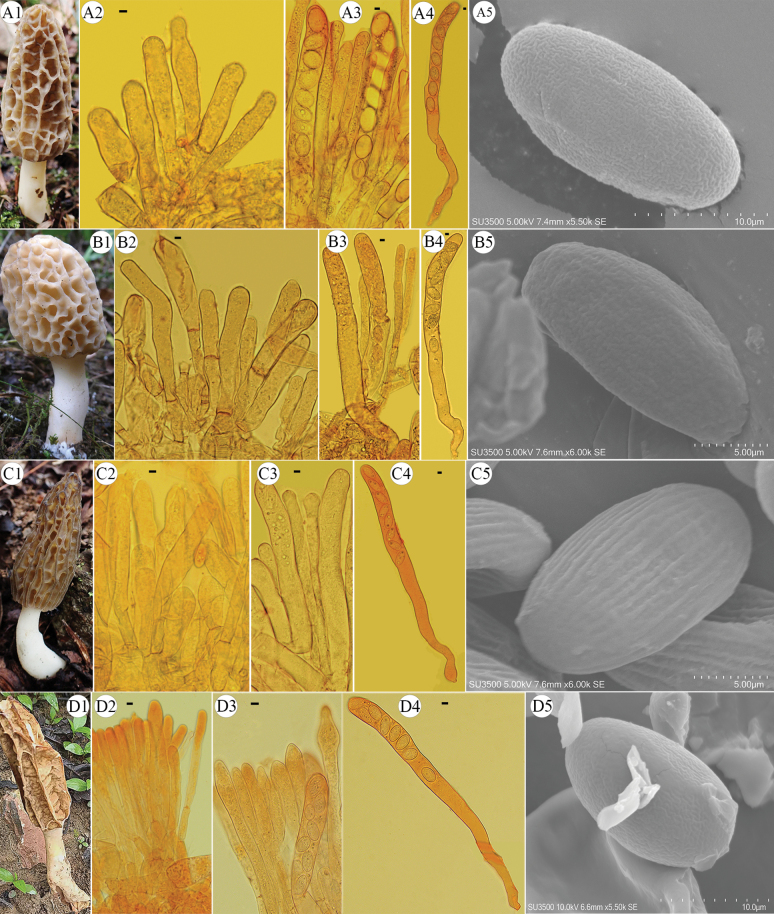
Morphological characteristics of new species, *Morchellauniversitatis*, *Morchellamontana*, *Morchelladiversa*, and one new record, *Morchellanipponensis*. A. Macro- and micro-morphological character of *Morchellauniversitatis* sp. nov. (FCNU1318). A1. Ascocarp. A2. Acroparaphyses. A3. Paraphyses. A4. Ascus. A5. SEM image of an ascospore, with irregular longitudinal and interconnecting transverse ornamentation. B. Macro- and micro-morphological characters of *Morchellamontana* sp. nov. (FCNU1206). B1. Ascocarp. B2. Acroparaphyses. B3. Paraphyses. B4. Ascus. B5. SEM image of an ascospore, with regular longitudinal ornamentation. C. Macro- and micro-morphological characters of *Morchelladiversa* sp. nov. (FCNU1247). C1. Ascocarp. C2. Acroparaphyses. C3. Paraphyses. C4. Ascus. C5. SEM image of an ascospore, with regular longitudinal ornamentation. D. Macro- and micro-morphological characters of *Morchellanipponensis* (FCNU1438). D1. Ascocarp. D2. Acroparaphyses. D3. Paraphyses. D4. Ascus. D5. SEM image of an ascospore, with irregular longitudinal and interconnecting transverse ornamentation. Scale bars: 5 μm (A2–A4, B2–B5, C2–C5, D2–D4); 10 μm (A5, D5).

##### Diagnosis.

Middle-appearing, small- to middle-sized morel, characterized by an oval or bluntly conical pileus with deep and dense or sparse (depending on individuals) pits in irregular sizes and shapes and primary vertical and transecting horizontal ridges. Pileus grayish at juvenility and yellowish at maturity. Stipe whitish to yellowish, of medium length, obviously wrinkled at the surface, glabrous, or finely mealy with yellowish granules. Paraphyses slender, sometimes slightly inflated at the apex. Usually occurring in subtropical broad-leaved mixed forest, associated with *Buxus* sp., *Kalopanax* sp., *Rheum* sp., *Cupressus* sp., and *Pinus* sp., sometimes beside weeds.

##### Type.

**China • 1**; Chongqing Municipality, Chengkou County. 26 Apr 2024, Q. Qin (FCNU1318—***holotype*** preserved in the Fungal Herbarium of Chongqing Normal University). GenBank: ITS = PV156389; *EF1-a* = PV227258; *RPB1* = PV227214; *RPB2* = PV175858.

##### Description.

Ascomata 123 mm high. Hymenophore 45 mm high; 47 mm wide at the widest point; irregularly campanulate; margin slightly undulate; yellow to light yellowish-brown; pitted and ridged, with 8–10 primary vertical ridges and some shorter, secondary vertical ridges, and transecting horizontal ridges, forming a honeycomb-like structure. Ridges finely tomentose; light yellow before full maturity. Pits asymmetrical, usually irregularly polygonal; mainly small to middle; in high dense; glabrous or finely tomentose; deep and irregular in outline and size; pale brownish yellow to yellowish brown. Stipe 78 mm high, 15 mm wide, clavate to subclavate, finely mealy with scattered whitish to yellowish granules. Context whitish; 1–2 mm thick in the hollow hymenophore; sometimes slightly chambered near the base. Sterile inner surface whitish and pubescent.

Ascospores (19.55-) 20.31–25.96 (-29.04) × (10.44-) 11.81–16.78(-17.57) μm (Q = 1.38–2.17, Qm = 1.62 ± 0.17), subelliptical to elliptical, with nearly irregular longitudinal and interconnecting transverse ornamentation; contents homogeneous. Asci cylindrical, eight-spored, (244.23-) 285.57–395.55 (-397.40) × (17.18-) 18.74–25.82 (-25.96) μm. Paraphyses, generally rounded to subclavate, septate, (149.11-) 153.29–201.79 (-217.93) × (10.96-) 11.09–17.22 (-17.38) μm, but difficult to measure their length. Elements on sterile ridges (63.86-) 77.17–106.42 (-106.75) × (13.51-) 13.99–21.23 (-23.09) μm. The number of ascospores in asci varies from two, four, five, six, seven, eight, nine, and ten.

##### Habitat.

Solitary or in clusters of two on the ground, occurring under *Rheum* sp.

##### Distribution.

This species is only known from China.

##### Other specimens examined.

**China • 1**; Chongqing Municipality, Chengkou County. 26 Apr 2024, Q. Qin (FCNU1325—***paratype*** preserved in the Fungal Herbarium of Chongqing Normal University). **China • 1**; Chongqing Municipality, Qianjiang District. 8 Apr 2024, Q. Qin (FCNU1368—***paratype*** preserved in the Fungal Herbarium of Chongqing Normal University). **China • 1**; Chongqing Municipality, Qianjiang District. 8 Apr 2024, Q. Qin (FCNU1381—***paratype*** preserved in the Fungal Herbarium of Chongqing Normal University).

##### Comments.

This morphologically distinct species corresponds to the previously reported phylospecies *Morchella* sp. Mes-19 determined by multi-gene phylogenetic analysis in Du et al. (2012a).

#### ﻿ Morchella montana

Taxon classificationAnimaliaPezizalesMorchellaceae

Q. Qin & X.H. Du sp. nov.

26254507-6932-5D32-86C6-7DEE29C62DFF

859402

Figs 2K, L , 9B

##### Etymology.

The specific epithet montana is derived from the Latin adjective montānus, -a, -um (“of the mountains”). It refers to the frequent occurrence of this species at high elevations and its type locality in Chongqing, which is widely known as “Mountain City” in China.

##### Diagnosis.

Middle- to late-appearing, middle-sized morel, characterized by an oval or bluntly conical pileus with deep, small, and very dense pits in irregular sizes and shapes, and with rich vertical and transecting horizontal ridges, yellowish at maturity. Stipe whitish to yellowish, middle length, glabrous or finely mealy with yellowish granules. Paraphyses slender, with slightly inflated at the upper middle place. Occurring under *Castanopsis* sp.

##### Type.

**China • 1**; Chongqing Municipality, Pengshui County. 9 Apr 2023, Q. Qin (FCNU1206—***holotype*** preserved in the Fungal Herbarium of Chongqing Normal University). GenBank: ITS = PV156371; *EF1-a* = PV227277; *RPB1* = PV227221; *RPB2* = PV175868.

##### Description.

Ascomata 82 mm high. Hymenophore 24 mm high; 38 mm wide at the widest point; ovoid with a conical apex; light yellowish-brown to beige; pitted and ridged, with 10–12 primary vertical ridges, some shorter secondary vertical ridges, and transecting horizontal ridges, forming a typical morel-like structure. Ridges thick, sometimes sharpened, finely tomentose or glabrous; grayish. Pits asymmetrical, relatively deep; primarily elongated vertically; glabrous or finely tomentose; pale yellowish or nearly whitish. Stipe cylindrical, slightly inflated in the lower half; 58 mm high; 15 mm wide; glabrous or finely mealy with yellowish white granules; whitish to pale yellowish. Context whitish; 1–2 mm thick in the hollow hymenophore; sometimes slightly chambered near the base. Sterile inner surface whitish and pubescent.

Ascospores measuring (13.65-) 15.08–20.41 (-20.64) × (8.74-) 9.15–13.20 (-13.73) μm (Q = 1.32–1.95, Qm = 1.63 ± 0.15), elliptical to ovoid, with regular longitudinal ornamentation; contents homogeneous. Asci cylindrical, eight-spored, (201.40-) 218.80–310.13 (-312.45) × (17.09-) 17.45–23.98 μm. Paraphyses, cylindrical in shape, apices usually rounded or subclavate but occasionally clavate, septate, (103.80-) 105.75–149.21 (-155.62) × (6.62-) 7.15–15.83 (-16.91) μm, but difficult to measure their length. Elements on sterile ridges (71.16-) 72.25–124.97 (-133.54) × (9.69-) 10.11–18.38 (-19.56) μm, septate. The number of ascospores in asci observed to vary between one and eight.

##### Habitat.

Solitary on the ground under *Castanopsis* sp.

##### Distribution.

This species is only recorded from southwestern China.

##### Other specimens examined.

**China • 1**; Chongqing Municipality, Wuxi County. 27 Apr 2024, Q. Qin (FCNU1328—***paratype*** preserved in the Fungal Herbarium of Chongqing Normal University).

##### Comments.

This morphologically distinct species corresponds to the previously reported phylospecies *Morchella* sp. Mes-20 determined by multi-gene phylogenetic analysis in Du et al. (2012a).

#### ﻿ Morchella diversa

Taxon classificationAnimaliaPezizalesMorchellaceae

Q. Qin & X.H. Du sp. nov.

ADF10226-BC0F-5ED1-8AF2-30B6DE3734DA

859403

Figs 2G , 3 , 9C

##### Etymology.

The specific epithet diversa is derived from the Latin adjective diversus, meaning “different, varied, divers.” It refers to the diverse vegetation found in the habitats where this species grows.

##### Diagnosis.

Early- to middle-appearing, small- to middle-sized morel, characterized mainly by a conical (sometimes oval or bluntly conical) pileus with shallow to deep, large and dominantly vertical pits of irregular sizes and shapes, and with primary vertical and secondary transecting horizontal tomentose ridges. Ridges dark brown to black at maturity. The color of pielus varying from grayish at juvenility to dark brown at maturity, sometimes light brown or yellowish, easily affected by environmental conditions. Stipe whitish to yellowish, short to middle length, finely mealy with whitish granules. Paraphyses slender, without obviously inflated at the apex. Occurring in subtropical broad-leaved or mixed forests, associated with Bamboo, *Quercus* sp., *Pinus* sp., *Quercus* sp., and *Daphniphyllum* sp.

##### Type.

**China • 1**; Chongqing Municipality, Kaizhou District. 27 Mar 2024, Q. Qin (FCNU1247—***holotype*** preserved in the Fungal Herbarium of Chongqing Normal University). GenBank: ITS = PV156381; *EF1-a* = PV227299; *RPB1* = PV211899; *RPB2* = PV175817.

##### Description.

Ascomata 68 mm high. Hymenophore 53 mm high; 23 mm wide at the widest point; conical or lanceolate; light brown with slightly glossy; pitted and ridged, with approximately 12–15 vertical ridges and numerous horizontal and transecting ridges; forming a honeycomb-like structure. Ridges thick, sometimes sharpened; obviously tomentose; dark brown to gray. Pits primarily elongated vertically; irregular in outline and size, relatively deep; finely tomentose; pale brown. Stipe slightly inflated at the base, 15 mm high; 11 mm wide; obviously mealy with whitish granules; pure white. Context whitish; 1–3 mm thick in the hollow hymenophore; sometimes slightly chambered near the base. Sterile inner surface whitish and pubescent.

Ascospores measuring (14.33-) 15.04–20.32 (-21.37) × (6.76-) 7.75–10.53 (-11.90) μm (Q = 1.70–2.57, Qm = 1.99 ± 0.19), elliptical to ovoid, with regular, obviously protuberant longitudinal ornamentation; contents homogeneous. Asci cylindrical, eight-spored, (229.47-) 234.84–263.07 (-263.63) × (15.34-) 15.45–18.28 (-19.99) μm. Paraphyses, cylindrical in shape, apices generally rounded but occasionally subclavate to clavate, septate, (109.84-) 114.58–148.93 (-159.68) × (8.72-) 9.13–13.93 (-15.20) μm, but difficult to measure their length. Elements on sterile ridges (74.00-) 78.06–122.55 (-123.38) × (11.62-) 11.94–19.85 (-20.66) μm. The number of ascospores in asci observed to vary among five, seven, and eight.

##### Habitat.

Occurring solitary or in clusters of two on the ground under *Pinus* sp.

##### Distribution.

This species is only recorded from China and Japan.

##### Other specimens examined.

**China • 1**; Chongqing Municipality, Kaizhou District. 27 Mar 2024, Q. Qin (FCNU1248—***paratype*** preserved in the Fungal Herbarium of Chongqing Normal University).

##### Comments.

This morphologically distinct species corresponds to the previously reported phylospecies *Morchella* sp. *Mel*-21 determined by multi-gene phylogenetic analysis in O’Donnell et al. (2011).

#### ﻿ Morchella nipponensis

Taxon classificationAnimaliaPezizalesMorchellaceae

Clowez, Izumi, Lamiable, Shibakusa & P. Alvarado, Mycoscience 63(6): 282 (2022).

51F9C116-5112-5B98-A134-23F26346081E

Figs 2D , 9D

##### Description.

Ascomata lanceolate; attached to the stipe with a small sinus; longitudinally pitted and ridged, with primary vertical ridges and some shorter, secondary vertical ridges, and transecting horizontal ridges. Ridges are slender and finely tomentose; yellow-orange or locally becoming orange brown. Large pits, asymmetrical, deep, flat, and irregular in outline and size; glabrous or finely tomentose; primarily elongated vertically. Stipe cylindrical; slightly swollen in the lower half; glabrous or finely mealy with orange-yellow granules. Context whitish; 1–2 mm thick in the hollow hymenophore; thickened and slightly chambered near the base. Sterile inner surface whitish and pubescent.

Ascospores measuring (15.40-) 15.53–21.34 (-21.61) × (9.13-) 9.56–12.82 (-12.92) μm (Q = 1.19–2.05, Qm = 1.76 ± 0.21), elliptical to ovoid, with nearly irregular longitudinal and interconnecting transverse ridges; contents homogeneous. Asci cylindrical, eight-spored, frequently (248.50-) 266.25–340.92 (-346.30) × (16.98-) 17.11–24.31 (-25.21) μm. Paraphyses, cylindrical in shape, apices generally rounded but occasionally subclavate to clavate; septate, (123.28-) 142.03–365.04 (-367.07) × (11.04-) 12.01–20.75 (-22.92) μm, but difficult to measure their length. Elements on sterile ridges (120.04-) 121.05–185.19 (-195.97) × (10.52-) 11.57–14.9 (-16.31) μm, difficult to measure their length. The number of ascospores in asci observed to vary from two to eight.

##### Habitat.

Solitary on the ground around the base of *Artemisia* sp.

##### Distribution.

This species is only known from Chongqing, China, and Japan.

##### Other specimens examined.

**China • 1**; Chongqing Municipality, Wuxi County, alt. 1697 m, 10 May 2024, Q. Qin (FCNU1436—preserved in the Fungal Herbarium of Chongqing Normal University). **China • 1**; Chongqing Municipality, Wuxi County, alt. 1697 m, 10 May 2024, Q. Qin (FCNU1437—preserved in the Fungal Herbarium of Chongqing Normal University). **China • 1**; Chongqing Municipality, Wuxi County, alt. 1697 m, 10 May 2024, Q. Qin (FCNU1438—preserved in the Fungal Herbarium of Chongqing Normal University).

##### Comments.

*Morchellanipponensis* is a record new to China. The presence of this species in Chongqing extends its distribution range from Japan to China. That the current disjunct distributions of *M.nipponensis* in China and Japan are attributed to relatively recent long-distance dispersals or human introductions remains an open question. The macromorphology of the Chinese collection is in accordance with the descriptions of typical *M.nipponensis*, which was originally described from Japan (Clowez et al. 2022). Microscopically, the ascospores of Chinese collections are smaller than those reported from Japan, (18-) 20–23.5 (-24) × (10.5-) 11.5–12.5 (-13) µm, but asci are larger than those from Japan by Clowez et al. (2022). The multi-gene sequences of *M.nipponensis* generated from the Chinese collections are closely related to that generated from the Japan collection, and the phylogenetic position of this species is well-settled (Fig. 7).

## ﻿Discussion

### ﻿Unexpected high species diversity of *Morchella* in Chongqing

Multi-gene phylogenetic analyses led to the discovery of 16 *Morchella* species in Chongqing, including 13 in the Esculenta clade and three in the Elata clade (Figs 1–3), based on an eight-year survey during the fruiting season in Chongqing from 2017 to 2024. So far, 37 phylospecies have been previously reported in China, including 21 from the Esculenta clade and 16 from the Elata clade, based on the GCPSR criterion (Du et al. 2012a, 2014, 2015, 2019). With the discovery of two newly identified species and one new record in Chongqing from this study, the number of *Morchella* species confirmed to be distributed in China has increased to 40. Interestingly, given that Chongqing covers an area of approximately 82,400 square kilometers, compared to China’s 9,600,000 square kilometers (Li 2024; Zhang et al. 2024), the species density of *Morchella* in Chongqing is approximately 50 times higher than the national average.

East Asia, particularly China, has been acknowledged as a center of modern species diversity of *Morchella* (Du et al. 2012a, 2014, 2015). This is further substantiated by the current discovery of rich species diversity in Chongqing. During the Quaternary glaciation, Chongqing experienced minimal impact, protected by the Qinling Mountains (Gong and Chen 2006; Zhang et al. 2014; Ni et al. 2018). This protection allowed for the persistence of extensive mountainous refugia, which have played a critical role in conserving regional biodiversity, such as floras (Liu 2000; Fan et al. 2022). The components of Chongqing’s mountainous refugia likely contribute significantly to the region’s high species diversity of *Morchella*. The rich species density observed in Chongqing suggests that this region could serve as a hotspot for *Morchella* species diversity in China. The findings not only highlight the importance of regional studies in uncovering the diversity of *Morchella* but also shed light on their evolutionary history and biogeographical patterns.

### ﻿The Sino-Japanese and Sino-Himalayan Forest subkingdoms shaping species diversity and distribution pattern of *Morchella* in Chongqing

Chongqing is located at the intersection of the Sino-Japanese and Sino-Himalayan Forest subkingdoms (Ding et al. 2013) but is widely regarded as possessing significant components of the Sino-Japanese Forest subkingdom (Liu 2000). Among all the *Morchella* species previously identified in China, 24 species were found in the Sino-Japanese Forest subkingdom, 18 in the Sino-Himalayan Forest subkingdom, 7 in the Eurasia Forest subkingdom, and 4 in the Qinghai–Xizang Plateau subkingdom (Du et al. 2012a, 2014, 2015, 2019). The Sino-Japanese and Sino-Himalayan Forest subkingdoms are considered the primary distribution areas for *Morchella* species in China (Du et al. 2012a, 2015). The former serves as the principal distribution center for species in the Esculenta clade, which are commonly found in broadleaved forests, while the latter is associated with species in the Elata clade, which are typically found in coniferous forests (Du et al. 2012a, 2015).

Thirteen species in the Esculenta clade and three species in the Elata clade were identified in Chongqing (Figs 6, 7). According to the recorded dominant vegetation for the 16 species in Chongqing (Table 1), most species in the Esculenta clade grew under broadleaved or mixed forests, while the three species in the Elata clade mainly grew under coniferous forests, such as *Pinus* sp. In addition, given the dominance of broadleaf forests in Chongqing (Liu 2000; Ni et al. 2018; Zhang 2019), the predominance of species from the Esculenta clade in Chongqing is consistent with the previous opinion that the preferred habitats of *Morchella* species are correlated with vegetation type to some extent (Du et al. 2012a, 2014, 2019). Notably, among the 16 species in Chongqing, *M.diversa* and *M.universitatis* occur in a broader range of habitats characterized by diverse dominant vegetation, such as bamboo, *Quercus* sp., *Pinus* sp., and *Daphniphyllum* sp. for the former (Table 1). Interestingly, consistent with their ecological breadth, these two species are also the most widely distributed in the Elata and Esculenta clades, respectively (Fig. 1). This indicates that *Morchella* species with greater habitat adaptability tend to possess broader geographic ranges.

Furthermore, *M.nipponensis* and *Morchella* sp. Mes-12 have previously been recorded exclusively in Japan (Du et al. 2012a; Clowez et al. 2022). The new records of both species in Chongqing may be associated with their preferred habitats within the Sino-Japanese Forest subkingdom. However, it remains unclear whether the current disjunct distributions are the result of relatively recent transoceanic long-distance dispersal or human introductions of horticultural plants with soil containing ascospores or mycelia.

### ﻿Effect of high summer temperature and habitat on species diversity of *Morchella* in Chongqing

Terry et al. (2011) found that rising temperatures adversely affect the composition of mammal communities, decreasing species richness and favoring heat-tolerant species while threatening less adaptable species. Urban (2015) suggested that up to 30% of species are at risk of extinction with every 1 °C increase in temperature. Temperature is also considered a major determinant of the distribution patterns of fungi (Boddy et al. 2013; Luo et al. 2021). Chongqing is well known as a “Furnace City” in China, with extremely high summer temperatures. For example, in 2024, the highest temperature in Chongqing reached 43.6 °C, and temperatures above 40 °C lasted from July to the end of September (Fig. 4B; Suppl. material 1: table S4). Investigating temperature-sensitive fungi in Chongqing is helpful for understanding local biodiversity and fungal adaptability in response to high temperature.

*Morchella* species are commonly known as cold-preferring fungi, which predominantly fruit in late spring (O’Donnell et al. 2011; Du et al. 2015; Loizides et al. 2021). The remarkably rich species diversity of *Morchella* uncovered in Chongqing suggests that it is not strongly affected by the region’s high summer temperatures. High temperatures in Chongqing primarily occur from June to August (Tang and Gao 2003; Wu et al. 2024), during which *Morchella* mainly undergoes vegetative growth through its mycelial networks. Though the quality of mycelial growth is closely linked to fruiting body development (Foulongne-Oriol et al. 2014; Salmones et al. 2018), the mycelia of *Morchella* grow primarily in soil and humus during summer, which reduces exposure to extreme heat. Moreover, compared to the vegetative mycelial stage, climatic variables are more critical for initiating fruiting and sporocarp development (Boddy et al. 2013). During the fruiting season, local temperatures in Chongqing are generally favorable for sporocarp development (Fig. 4B), despite a compressed fruiting period caused by subsequent rapid warming. This growth strategy helps *Morchella* species endure high summer temperatures in Chongqing.

On the other hand, Chongqing is also well known as the “Mountain City.” According to Barry (2008), temperature on mountains decreases by 0.6 °C for every 100 m increase in altitude. Habitat altitude data from *Morchella* specimens collected in Chongqing range from 280 m to 2,150 m, with 90% collected above 800 m (Table 1). According to annual maximum temperature records from 2017 to 2024 across the 13 districts and counties investigated (Fig. 4A; Suppl. material 1: table S3), actual temperatures at the collection sites were likely 0.3–10 °C lower than those reported by local meteorological stations. Among all the districts and counties investigated, Wuxi stands out as the center of *Morchella* species diversity in Chongqing, harboring eight species (Fig. 1), most of which were collected at altitudes ranging from 1,300 m to 2,194 m. Although local temperatures in Wuxi are generally higher than those in other regions (Fig. 4A), its diverse mountainous habitats and complex topography provide relatively favorable conditions for *Morchella* to thrive. Strangely, Youyang County consistently recorded the lowest temperatures among all 13 districts and counties from 2017 to 2024 (Fig. 4A), despite being located in the Wuling Mountains, yet it harbored only two species (Fig. 1). Overall, we presume that the combination of diverse mountainous habitats, a favorable fruiting period in spring for these cold-preferring fungi, and rich vegetation contributes to the unexpectedly high species diversity of *Morchella* in the “Furnace City,” Chongqing. However, this raises another question regarding the diversity of non-cold-preferring fungi in such areas, which requires further research.

## ﻿Conclusion

This study presents a significant advancement in our understanding of true morel diversity in Chongqing, identifying a total of 16 species, including six new species and one new record from China. The unexpected species diversity of *Morchella* uncovered in Chongqing has been previously overlooked due to the region’s high temperatures. The distinct species diversity observed within the Elata and Esculenta clades in Chongqing further underscores the correlation between species distribution in *Morchella* and their preferred vegetation, highlighting the importance of ecological factors in the evolutionary history of *Morchella*. This research enriches our understanding of local biodiversity in challenging environments.

## Supplementary Material

XML Treatment for Morchella chinensis

XML Treatment for Morchella huoguo

XML Treatment for Morchella eoa

XML Treatment for Morchella universitatis

XML Treatment for Morchella montana

XML Treatment for Morchella diversa

XML Treatment for Morchella nipponensis

## ﻿Additional information

### Use of AI

No use of AI was reported.

### Adherence to national and international regulations

All the fungal strains used in this study have been legally obtained, respecting the Convention on Biological Diversity (Rio Convention).

### Author ORCIDs

Qin Qin https://orcid.org/0009-0002-6655-5909

Wen-Shu Hu https://orcid.org/0009-0000-5466-8522

Zhong-Dong Yu https://orcid.org/0000-0002-9625-1239

Xia Guo https://orcid.org/0000-0002-8566-2649

Xi-Hui Du https://orcid.org/0000-0001-8113-5799

### Data availability

The newly generated sequences in this study have been uploaded to GenBank, and the corresponding accession numbers are provided in Table 1.

## Supplementary materials

Supplementary material 1 Detailed information of retrieved sequences from GenBank Data type docx https://binary.pensoft.net/file/1392909This dataset is made available under the Open Database License (http://opendatacommons.org/licenses/odbl/1.0/). The Open Database License (ODbL) is a license agreement intended to allow users to freely share, modify, and use this Dataset while maintaining this same freedom for others, provided that the original source and author(s) are credited.Qin Qin, Yan-Fei Teng, Wen-Shu Hu, Jing-Yi Wei, Zhong-Dong Yu, Ping Du, Xiao-Yan Zhang, Xia Guo, Meng-Qian Chen, Wei Wei, Xi-Hui Du
