# Supplementary material for: ﻿Unexpected richness and distinct patterns of Morchella (Ascomycota) species diversity in Chongqing, a notable “Furnace City”: unveiling rich diversity in hot regions
Source: IMA Fungus. 2025 Aug 12;16:e152685. doi: 10.3897/imafungus.16.152685 (PMC12365677; doi:10.3897/imafungus.16.152685)
Supplement: Supplementary material 1 — Detailed information of retrieved sequences from GenBank [file imafungus-16-e152685-s001.docx]

**Unexpected richness and distinct patterns of *Morchella* species diversity in Chongqing, a notable ‘Furnace City’:**

**unveiling rich diversity in hot regions**

Qin Qin^1^, Yan-Fei Teng^1^, Wen-Shu Hu^1^, Jing-Yi Wei^2^, Zhong-Dong Yu^3^, Ping Du^4^, Xiao-Yan Zhang^1^, Xia Guo^1^, Meng-Qian Chen^1^, Wei Wei^1^, Xi-Hui Du^1*^

^1^ College of Life Sciences, Chongqing Normal University, Chongqing 401331, China

^2^ Chongqing Academy of Agricultural Sciences, Chongqing 401329, China

^3^ College of Forestry, Northwest A&F University, Yangling 712100, Shaanxi, China

^4^ School of Advanced Agriculture and Bioengineering, Yangtze Normal University, Chongqing 408100, China

^*^Correspondence:

Xi-Hui Du

duxihuimorel@outlook.com

**Supplemental Table S1.** Detailed information on the retrieved sequences of species in Elata Clade used in this study.

| Species | Voucher | Location | GenBank accession number | | | | Reference |
| --- | --- | --- | --- | --- | --- | --- | --- |
|  |  |  | ITS | *EF1-a* | *RPB1* | *RPB2* |  |
| *Morchella exuberans* | HKAS62866 | China | JQ321880 | JQ321848 | JQ321944 | JQ321976 | Du et al. 2012a |
|  | HKAS62867 | China | JQ321881 | JQ321849 | JQ321945 | JQ321977 | Du et al. 2012a |
| *M. importuna* | HKAS62868 | China | JQ321874 | JQ321842 | JQ321938 | JQ321970 | Du et al. 2012a |
|  | HKAS62871 | Germany | JQ321903 | JQ321871 | JQ321967 | JQ321999 | Du et al. 2012a |
| *M. owerii* | FCNU1025 | China | MK321846 | MK321864 | MK321852 | MK321858 | Du et al. 2019 |
|  | FCNU1026 | China | MK321847 | MK321865 | MK321853 | MK321859 | Du et al. 2019 |
| *M. eximia* | M833 | Canada | JQ723042 | GU550997 | GU551080 | GU551121 | Du et al. 2012a; O'Donnell et al. 2011 |
|  | HKAS62863 | China | JQ321901 | JQ321869 | JQ321965 | JQ321997 | Du et al. 2012a |
| *M. sextelata* | M88 | USA | JQ723039 | GU551546 | GU551644 | / | Du et al. 2012a; O'Donnell et al. 2011 |
|  | HKAS62872 | China | JQ321877 | JQ321845 | JQ321941 | JQ321973 | Du et al. 2012a |
| *M. purpurascens* | HT297 | Turkey | JN085111 | JN085055 | JN085171 | JN085227 | Taşkın et al. 2012 |
|  | M476 | China | GU551426 | GU551389 | GU551463 | GU551505 | O'Donnell et al. 2011 |
| *M. eohespera* | M215 | Sweden | GU551404 | GU551367 | GU551441 | GU551478 | O'Donnell et al. 2011 |
|  | HKAS62873 | China | JQ321878 | JQ321846 | JQ321942 | JQ321974 | Du et al. 2012a |
| *M. laurentiana* | 13.05.14AV01 | Canada | KT819374 | KT819385 | KT819351 | KT819362 | Voitk et al.2014 |
|  | 10.05.19AV02 | Canada | KT819376 | KT819387 | KT819353 | KT819364 | Voitk et al.2014 |
| *M. hispaniolensis* | M374 | Dominican Republic | MH014725 | GU551554 | GU551652 | GU551484 | Baroni et al. 2018; O'Donnell et al. 2011 |
| *M. kaibabensis* | TAC-1376 | USA | MH014727 | MH014721 | MH014732 | MH014737 | Baroni et al. 2018 |
|  | TAC-1708 | USA | MH014728 | MH014722 | MH014733 | MH014738 | Baroni et al. 2018 |
| *M. brunnea* | M35 | Canada | GU551415 | GU551378 | GU551452 | GU551492 | O'Donnell et al. 2011 |
|  | M431 | USA | GU551414 | GU551377 | GU551451 | GU551491 | O'Donnell et al. 2011 |
| *M. fekeensis* | HT401 | Turkey | JN085114 | JN085058 | JN085174 | JN085230 | Taşkın et al. 2012 |
|  | HT510 | Turkey | JN085133 | JN085077 | JN085193 | JN085249 | Taşkın et al. 2012 |
| *M. magnispora* | HT470 | Turkey | JN085122 | JN085066 | JN085182 | JN085238 | Taşkın et al. 2012 |
|  | HT471 | Turkey | JN085123 | JN085067 | JN085183 | JN085239 | Taşkın et al. 2012 |
| *M. arbutiphila* | HT193 | Turkey | JN085141 | JN085085 | JN085201 | JN085257 | Taşkın et al. 2012 |
| *M. septentrionalis* | M9 | USA | JQ723064 | GU551556 | GU551654 | GU551487 | Du et al. 2012a; O'Donnell et al. 2011 |
| *M. pulchella* | HT472 | Turkey | JN085124 | JN085068 | JN085184 | JN085240 | Taşkın et al. 2012 |
| *M. confericola* | HT106 | Turkey | JN085140 | JN085084 | JN085200 | JN085256 | Taşkın et al. 2012 |
|  | HT479 | Turkey | JN085127 | JN085071 | JN085187 | JN085243 | Taşkın et al. 2012 |
| *M. australiana* | M338 | Australia | KC753472 | KC753468 | KC753475 | KC753480 | Elliott et al. 2014 |
|  | T35077 | Australia | KC753470 | KC753466 | KC753477 | KC753478 | Elliott et al. 2014 |
| *M. kakiicolor* | M288 | Canary Island | JQ723065 | GU551567 | GU551665 | GU551527 | Du et al. 2012a; O'Donnell et al. 2011 |
| *M. eximioides* | M231 | Sweden | GU551428 | GU551391 | GU551465 | GU551508 | O'Donnell et al. 2011 |
|  | HKAS62883 | China | JQ321898 | JQ321866 | JQ321962 | JQ321994 | Du et al. 2012a |
| *M. angusticeps* | M304 | USA | JQ723055 | GU551560 | GU551658 | GU551707 | Du et al. 2012a; O'Donnell et al. 2011 |
|  | M65 | USA | GU551433 | GU551396 | GU551470 | GU551516 | O'Donnell et al. 2011 |
| *M. confusa* | FCNU1027 | China | MK321848 | MK321866 | MK321854 | MK321860 | Du et al. 2019 |
|  | FCNU1028 | China | MK321849 | MK321867 | MK321855 | MK321861 | Du et al. 2019 |
| *M. synderi* | M299 | USA | GU551413 | GU551376 | GU551450 | GU551490 | O'Donnell et al. 2011 |
|  | M433 | USA | GU551425 | GU551388 | GU551462 | GU551503 | O'Donnell et al. 2011 |
| *M. mediterraneensis* | HT448 | Turkey | JN085118 | JN085062 | JN085178 | JN085234 | Taşkın et al. 2012 |
|  | HT520 | Turkey | JN085135 | JN085079 | JN085195 | JN085251 | Taşkın et al. 2012 |
| *M. dunalii* | HT436 | Turkey | JN085117 | JN085061 | JN085177 | JN085233 | Taşkın et al. 2012 |
|  | HT539 | Turkey | JN085136 | JN085080 | JN085196 | JN085252 | Taşkın et al. 2012 |
| *M. semilibera* | M144 | Netherlands | JQ723024 | GU550993 | GU551075 | GU551116 | Du et al. 2012a; O'Donnell et al. 2011 |
|  | M162 | Czech Republic | JQ723023 | GU551552 | GU551650 | GU551699 |  |
| *M. punctipes* | M28 | USA | JQ723026 | GU550978 | GU551060 | GU551101 |  |
|  | M64 | USA | JQ723027 | GU550981 | GU551063 | GU551104 |  |
| *M. populiphila* | M89 | USA | JQ723032 | GU551545 | GU551643 | GU551692 |  |
|  | M903 | USA | JQ723033 | / | JQ670120 | JQ670131 |  |
| *M. tridentina* | M17 | Canada | JQ723020 | GU550989 | GU551071 | GU551112 |  |
|  | M84 | USA | JQ723022 | GU551547 | GU551645 | GU551694 |  |
| *M. odonnellii* | FCNU1023 | China | MK321850 | MK321868 | MK321856 | MK321862 | Du et al. 2019 |
|  | FCNU1024 | China | MK321851 | MK321869 | MK321857 | MK321863 | Du et al. 2019 |
| *M. tomentosa* | M105 | USA | JQ723016 | GU550987 | GU551069 | GU551110 | Du et al. 2012a; O'Donnell et al. 2011 |
|  | M46 | USA | JQ723017 | GU550983 | GU551065 | GU551106 |  |
| *M. disparilis* | ML5143MD2 | Cyprus | / | KU865051 | KU865038 | KU865043 | Loizides et al. 2016 |
|  | ML51481MD | Cyprus | KU865031 | KU865056 | KU865037 | KU865048 | Loizides et al. 2016 |
| *M. aysenina* | UDEC-78 | Chile | MN355527 | MN611979 | MN602599 | MN611966 | Machuca et al. 2021 |
|  | UDEC-33 | Chile | MN355528 | MN611980 | MN602600 | MN611967 | Machuca et al. 2021 |
| *M. helvetica* | M19-7 | Switzerland | OR482719 | OR667835 | PP598875 | PP598876 | Cravero et al. 2024 |
|  | M19-4 | Switzerland | OR482716 | OR667832 | PP598873 | PP598874 | Cravero et al. 2024 |
| 1. *diversa*   (*Morchella* sp. *Mel*-21) | M225 | Japan | JN085156 | JN085099 | GU551657 | GU551507 | Taşkın et al. 2012;  O'Donnell et al. 2011 |
|  | HKAS62880 | China | JQ321882 | JQ321850 | JQ321946 | JQ321978 | Du et al. 2012a |
| *Morchella* sp*. Mel*-8 | M86 | USA | JQ723045 | GU551548 | GU551646 | GU551695 | Du et al. 2012a; O'Donnell et al. 2011 |
| *Morchella* sp. *Mel*-13 | HT508 | Turkey | JN085131 | JN085075 | JN085191 | JN085247 | Taşkın et al. 2012 |
|  | HKAS62893 | China | JQ321888 | JQ321856 | JQ321952 | JQ321984 | Du et al. 2012a |
| *Morchella* sp. *Mel*-14 | HKAS62885 | China | JQ321887 | JQ321855 | JQ321951 | JQ321983 | Du et al. 2012a |
|  | HKAS62886 | China | JQ321891 | JQ321859 | JQ321955 | JQ321987 | Du et al. 2012a |
| *Morchella* sp. *Mel*-17 | M315 | Bulgaria | JQ723057 | GU551561 | GU551659 | GU551708 | Du et al. 2012a; O'Donnell et al. 2011 |
| *Morchella* sp. *Mel*-23 | M495 | Norway | JN085153 | GU551381 | GU551455 | GU551495 | Taşkın et al. 2012;  O'Donnell et al. 2011 |
|  | M542 | Denmark | JQ723063 | GU551562 | GU551660 | GU551709 | Du et al. 2012a; O'Donnell et al. 2011 |
| *Morchella* sp*. Mel*-33 | HKAS62874 | China | JQ321893 | JQ321861 | JQ321957 | JQ321989 | Du et al. 2012a |
| *Morchella* sp. *Mel*-34 | HKAS62877 | China | JQ321896 | JQ321864 | JQ321960 | JQ321992 | Du et al. 2012a |
| *Morchella* sp. *Mel*-37 | CIEFAP5 | Argentina | KJ439678 | KJ569626 | KJ569594 | KJ569620 | Pildain et al. 2014 |
|  | CIEFAP71 | Argentina | KJ439673 | KJ569630 | KJ569596 | KJ569624 | Pildain et al. 2014 |
| *Morchella* sp. *Mel*-38 | ALV3206 | Cyprus | KU865009 | KU865050 | KU865040 | KU865042 | Loizides et al. 2016 |

**Supplemental Table S2.** Detailed information on the retrieved sequences of species in Esculenta Clade used in this study.

| Species | Voucher | Locality | GenBank Accession Number | | | | Reference |
| --- | --- | --- | --- | --- | --- | --- | --- |
|  |  |  | ITS | *EF1-a* | *RPB1* | *RPB2* |  |
| *Morchella esculenta* | HKAS59167 | China | JQ322074 | JQ322033 | JQ322156 | JQ322197 | Du et al. 2012a |
|  | HKAS59168 | China | JQ322075 | JQ322034 | JQ322157 | JQ322198 | Du et al. 2012a |
| *M. fluvialis* | HT519 | Turkey | JQ723096 | JN085291 | JN085338 | JN085388 | Du et al. 2012a;  Taşkın et al. 2012 |
| 1. *yangii* | FCNU1011 | China | MK321874 | MK321922 | MK321890 | MK321906 | Du et al. 2019 |
|  | FCNU1014 | China | MK321877 | MK321925 | MK321893 | MK321909 | Du et al. 2019 |
| 1. *yishuica* | FCNU1017 | China | MK321882 | MK321930 | MK321898 | MK321914 | Du et al. 2019 |
|  | FCNU1018 | China | MK321879 | MK321927 | MK321895 | MK321911 | Du et al. 2019 |
| 1. *clivicola* | FCNU1019 | China | MK321870 | MK321918 | MK321886 | MK321902 | Du et al. 2019 |
|  | FCNU1020 | China | MK321875 | MK321923 | MK321891 | MK321907 | Du et al. 2019 |
| 1. *galilaea* | HT485 | Turkey | JQ723082 | JN085289 | JN085336 | JN085386 | Du et al. 2012a;  Taşkın et al. 2012 |
|  | HKAS55840 | China | JQ322049 | JQ322008 | JQ322131 | JQ322172 | Du et al. 2012a |
| *M. peruviana* | CIPHAM004 | USA | MH014708 | MH014705 | MH014716 | MH014719 | Baroni et al. 2018 |
| *M. gracilis* | M330 | Venezula | JQ723085 | GU551530 | GU551628 | GU551677 | Du et al. 2012a; O'Donnell et al. 2011 |
|  | M684 | Ecuador | JQ723086 | GU551148 | GU551260 | GU551316 |  |
| *M. palazonii* | FCNU1031 | China | MK321883 | MK321931 | MK321899 | MK321915 | Du et al. 2019 |
|  | FCNU1032 | China | MK321884 | MK321932 | MK321900 | MK321916 | Du et al. 2019 |
| *M. prava* | M38 | Canada | JQ723099 | GU551533 | GU551631 | GU551680 | Du et al. 2012a; O'Donnell et al. 2011 |
|  | M910 | USA | JQ723100 | GU551193 | GU551305 | GU551361 |  |
| *M. dunensis* | HT225 | Turkey | JQ723101 | JN085296 | JN085343 | JN085393 | Du et al. 2012a;  Taşkın et al. 2012 |
|  | HT337 | Turkey | JQ723102 | JN085281 | JN085331 | JN085378 |  |
| *M. ulmaria* | M239 | Canada | JQ723088 | GU551151 | GU551263 | GU551319 | Du et al. 2012a; O'Donnell et al. 2011 |
|  | M70 | USA | JQ723089 | GU551532 | GU551630 | GU551679 |  |
| *M. americana* | M205 | USA | JQ723078 | GU551150 | GU551262 | GU551318 |  |
|  | M78 | USA | JQ723068 | GU551157 | GU551269 | GU551325 |  |
| *M. sceptriformis* | M780 | USA | JQ723111 | GU551171 | GU551283 | GU551339 |  |
|  | M887 | USA | JQ723112 | GU551187 | GU551299 | GU551355 |  |
| *M. diminutiva* | M42 | USA | JQ723104 | GU551158 | GU551270 | GU551326 |  |
|  | M856 | USA | JQ723105 | GU551183 | GU551295 | GU551351 |  |
| *M. steppicola* | M512 | Hungary | JQ723119 | / | GU551100 | GU551362 |  |
|  | M635 | Slovakia | JQ723120 | GU551543 | GU551641 | GU551690 |  |
| *M. nipponensis* | phc353 | Japan | OM230111 | OM179895 | OM179887 | OM179891 | Clowez et al. 2022 |
|  | phc362 | Japan | OM230110 | OM179894 | OM179886 | OM179890 | Clowez et al. 2022 |
| *M. anatolica* | ML914MAK1 | France | MT036043 | MT232952 | MT232936 | MT232944 | Loizides et al. 2021 |
|  | LIP:PhC233 | France | KM587974 | / | / | / | Richard et al. 2015 |
| *M. castaneae* | JMM0003 | Spain | MW888871 | / | / | / | Unpublished |
|  | JMM0001 | Spain | MW888870 | / | / | / | Unpublished |
| *M. eoa*  (*Morchella* sp. *Mes*-15) | HKAS62913 | China | JQ322046 | JQ322005 | JQ322128 | JQ322169 | Du et al. 2012a |
|  | HKAS62914 | China | JQ322047 | JQ322006 | JQ322129 | JQ322170 | Du et al. 2012a |
| *M. universitatis*  (*Morchella* sp. *Mes*-19) | HKAS56568 | China | JQ322062 | JQ322021 | JQ322144 | JQ322185 | Du et al. 2012a |
|  | HKAS56585 | China | JQ322063 | JQ322022 | JQ322145 | JQ322186 | Du et al. 2012a |
| *M. montana*  (*Morchella* sp. *Mes*-20) | HKAS55841 | China | JQ322050 | JQ322009 | JQ322132 | JQ322173 | Du et al. 2012a |
|  | HKAS55842 | China | JQ322051 | JQ322010 | JQ322133 | JQ322174 | Du et al. 2012a |
| *M. rufobrunnea* | M14 | USA | JQ723124 | GU550982 | GU551064 | GU551105 | Du et al. 2012a; O'Donnell et al. 2011 |
| *Morchella* sp. *Mes*-5 | M508 | France | / | GU551160 | JN085362 | JN085412 | Taşkın et al. 2012;  O'Donnell et al. 2011 |
| *Morchella* sp. *Mes*-6 | HMJAU5334 | China | JQ322082 | JQ322041 | JQ322164 | JQ322205 | Du et al. 2012a |
|  | HKAS59163 | China | JQ322073 | JQ322032 | JQ322155 | JQ322196 | Du et al. 2012a |
| *Morchella* sp. *Mes*-9 | HKAS59128 | China | JQ322070 | JQ322029 | JQ322152 | JQ322193 | Du et al. 2012a |
|  | M49 | Japan | JQ723094 | GU551536 | GU551634 | GU551683 | Du et al. 2012a; O'Donnell et al. 2011 |
| *Morchella* sp. *Mes*-10 | HKAS59141 | China | JQ322071 | JQ322030 | JQ322153 | JQ322194 | Du et al. 2012a |
| *Morchella* sp. *Mes*-12 | M50 | Japan | JQ723079 | GU551528 | GU551626 | GU551675 | Du et al. 2012a; O'Donnell et al. 2011 |
| *Morchella* sp. *Mes*-13 | HKAS25327 | China | JQ723080 | GU551541 | GU551639 | GU551688 | Du et al. 2012a |
|  | HKAS55922 | China | JQ322080 | JQ322039 | JQ322162 | JQ322203 | Du et al. 2012a |
| *Morchella* sp. *Mes*-21 | HKAS55921 | China | JQ322079 | JQ322038 | JQ322161 | JQ322202 | Du et al. 2012a |
|  | HKAS55920 | China | JQ322081 | JQ322040 | JQ322163 | JQ322204 | Du et al. 2012a |
| *Morchella* sp. *Mes*-22 | HKAS55917 | China | JQ322058 | JQ322017 | JQ322140 | JQ322181 | Du et al. 2012a |
|  | HKAS55919 | China | JQ322059 | JQ322018 | JQ322141 | JQ322182 | Du et al. 2012a |
| *Morchella* sp. *Mes*-23 | HKAS62911 | China | JQ322042 | JQ322001 | JQ322124 | JQ322165 | Du et al. 2012a |
|  | HKAS56571 | China | JQ322064 | JQ322023 | JQ322146 | JQ322187 | Du et al. 2012a |
| *Morchella* sp. *Mes*-24 | HMAS96865 | China | JQ322043 | JQ322002 | JQ322125 | JQ322166 | Du et al. 2012a |
| *Morchella* sp. *Mes*-25 | HKAS62861 | China | JQ322076 | JQ322035 | JQ322158 | JQ322199 | Du et al. 2012a |
|  | HKAS62862 | China | JQ322077 | JQ322036 | JQ322159 | JQ322200 | Du et al. 2012a |
| *Morchella* sp. *Mes*-26 | HKAS55912 | China | JQ322055 | JQ322014 | JQ322137 | JQ322178 | Du et al. 2012a |
|  | HKAS55913 | China | JQ322056 | JQ322015 | JQ322138 | JQ322179 | Du et al. 2012a |
| *Morchella* sp. *Mes*-27 | HKAS55896 | China | JQ322053 | JQ322012 | JQ322135 | JQ322176 | Du et al. 2012a |
|  | HKAS55897 | China | JQ322054 | JQ322013 | JQ322136 | JQ322177 | Du et al. 2012a |
| *Morchella* sp*. Mes*-28 | SN47 | Pakistan | OP297345 | OP918549 | OP918336 | OP918474 | Unpublished |
|  | ML51313MV | Cyprus | KU865020 | / | / | / | Loizides et al.2016 |

**Supplemental Table S3.** The annual maximum temperatures in 13 districts and counties of Chongqing from 2017 to 2024.

| District or county in Chongqing | Altitude of local meteorological station | Maximum temperature in each district or county/year | | | | | | | |
| --- | --- | --- | --- | --- | --- | --- | --- | --- | --- |
|  |  | 2017 | 2018 | 2019 | 2020 | 2021 | 2022 | 2023 | 2024 |
| Chengkou | 798.2 m | 38℃ | 37℃ | 37℃ | 35℃ | 37℃ | 38℃ | 36℃ | 40℃ |
| Fengdu | 290.5 m | 44℃ | 42℃ | 40℃ | 39℃ | 39℃ | 44℃ | 38℃ | 43℃ |
| Fuling | 372.8 m | 41℃ | 40℃ | 39℃ | 39℃ | 40℃ | 43℃ | 38℃ | 40℃ |
| Kaizhou | 216.5 m | 42℃ | 42℃ | 41℃ | 41℃ | 40℃ | 43℃ | 40℃ | 42℃ |
| Nanchuan | 698.8 m | 39℃ | 38℃ | 37℃ | 37℃ | 36℃ | 40℃ | 37℃ | 40℃ |
| Pengshui | 322.2 m | 41℃ | 40℃ | 40℃ | 36℃ | 40℃ | 42℃ | 40℃ | 41℃ |
| Qiangjiang | 786.9 m | 36℃ | 36℃ | 36℃ | 33℃ | 36℃ | 38℃ | 37℃ | 37℃ |
| Shapingba | 259.1 m | 42℃ | 42℃ | 40℃ | 40℃ | 41℃ | 44℃ | 39℃ | 41℃ |
| Shizhu | 632.3 m | 39℃ | 41℃ | 37℃ | 37℃ | 38℃ | 44℃ | 37℃ | 41℃ |
| Wulong | 406.9 m | 40℃ | 39℃ | 39℃ | 38℃ | 40℃ | 41℃ | 37℃ | 40℃ |
| Wuxi | 337.8 m | 40℃ | 42℃ | 40℃ | 39℃ | 41℃ | 44℃ | 40℃ | 42℃ |
| Youyang | 826.5 m | 36℃ | 34℃ | 35℃ | 31℃ | 34℃ | 37℃ | 33℃ | 37℃ |
| Yunyang | 297.2 m | 41℃ | 40℃ | 41℃ | 39℃ | 41℃ | 43℃ | 40℃ | 42℃ |

**Supplemental Table S4.** The monthly maximum temperatures recorded in Chongqing in 2024.

| Locality | Monthly maximum temperature in 2024 | | | | | | | | | | | |
| --- | --- | --- | --- | --- | --- | --- | --- | --- | --- | --- | --- | --- |
|  | Jan | Feb | Mar | Apr | May | Jun | Jul | Aug | Sep | Oct | Nov | Dec |
| Chongqing | 18℃ | 23℃ | 29℃ | 34℃ | 38℃ | 37℃ | 40℃ | 41℃ | 41℃ | 28℃ | 23℃ | 17℃ |
